# A Comparative Review on the Extraction, Antioxidant Content and Antioxidant Potential of Different Parts of Walnut (*Juglans regia* L.) Fruit and Tree

**DOI:** 10.3390/molecules24112133

**Published:** 2019-06-05

**Authors:** Ali Jahanban-Esfahlan, Alireza Ostadrahimi, Mahnaz Tabibiazar, Ryszard Amarowicz

**Affiliations:** 1 Nutrition Research Center, Tabriz University of Medical Sciences, Tabriz 5166-15731, Iran; a.jahanban@gmail.com (A.J.-E.); ostadrahimi@tbzmed.ac.ir (A.O.); mahnaz_tabibiazar@yahoo.com (M.T.); 2 Infectious and Tropical Diseases Research Center, Tabriz University of Medical Sciences, Tabriz 5166-15731, Iran; 3 Student Research Committee, Tabriz University of Medical Sciences, Tabriz 5166-15731, Iran; 4 Division of Food Sciences, Institute of Animal Reproduction and Food Research of the Polish Academy of Sciences, 10-468 Olsztyn, Poland

**Keywords:** antioxidants, antiradical activity, flavonoids, Juglandaceae, phenols, walnut

## Abstract

As a valuable tree nut, walnut is a well-known member of the Juglandaceae family. The fruit is made up of an outer green shell cover or husk, the middle shell which must be cracked to release the kernel, a thin layer known as skin or the seed coat, and finally, the kernel or meat. The nutritional importance of walnut fruit is ascribed to its kernel. The shell and husk are burned as fuel or discarded away as waste products. In the past two decades, the evaluation of the phenolic content and antioxidant activity of different parts of walnut has received great interest. In this contribution, the recent reports on the extraction and quantification of phenolic content from each part of the walnut tree and fruit using different solvents were highlighted and comparatively reviewed. The current review paper also tries to describe the antioxidant content of phenolic extracts obtained from different parts of the walnut tree and fruit. Additionally, the antioxidant and antiradical activities of the prepared extracts have also been discussed.

## 1. Introduction

Phenolic compounds are secondary metabolites present in different parts of all plants species [1,2]. They belong to a large and heterogeneous group of biologically active molecules, and their generation depends on different enzymes involved in various metabolic pathways [3]. The metabolism of these compounds is integrated into the biochemical and morphological regulatory patterns of plants [4]. Phenolic compounds are involved in the physiological mechanisms of fruit and tree development and growth, and they affect various characteristics of fruit pre- and post-harvest periods [5,6,7]. Phenolic compounds in plants are protective substances against numerous kinds of stresses that can be triggered by environmental conditions, pathogens, and injuries [1]. Such stresses are known to induce and influence the generation of phenolic compounds [8]. Therefore, phenolics play critical roles in the defense mechanisms of plants [9,10]. Moreover, phytochemical compounds, such as the phenolics are advantageous for human well-being. They can reduce the risk of cardiovascular and degenerative diseases by preventing the oxidative stress and oxidation of biological macromolecules [11,12,13]. It has been shown that phenolic compounds can scavenge free radicals and possess metal-chelating properties in addition to their described anti-cancer activities [14,15].

In recent years, much consideration has been given to characterizing the polyphenol content and evaluating the antioxidant activity of different plant-based materials, especially nuts because their regular consumption is associated with the reduction of some disease risk such as cancer and cardiovascular diseases (CVDs) [16]. The *Juglans* genus or particularly Juglandaceae family includes several species and is widely distributed all over the world. The Persian, English, common walnut or *Juglans regia* L. (*J. regia* L.), is its well-known member, comprises significant forms of deciduous trees detected mainly in temperate regions and commercially cultivated in Asia, western South America, the United States, and central and southern Europe [17]. Walnut is a large arbor tree species which is traditionally cultivated for its valuable wood and fruit, attaining the height of 25–35 m, and a trunk up to 2 m in diameter [18]. Walnut is an appreciated crop because the nut is very popular and widely consumed. Green walnut, shell, husk, kernel, bark, root, and leaves have been extensively used in the pharmaceutical and cosmetic industries [19]. *J. regia* L. is recognized as a rich source of different valuable chemicals because the kernel, fresh green fruit, husk, shell, skin, bark, leaves, and root are comprehensively studied to use in food, cosmetic and pharmaceutical industries. In this respect, all parts of walnut tree can be used as an excellent source of various compounds expressing antioxidant and antimicrobial potential, as well as antinociceptive, antihistaminic, antiulcer, antiasthmatic, antidiabetics, immunomodulator, antifertility, hepatoprotective, central nervous system stimulant, anti-inflammatory, wound healing, lipolytic, larvicidal, insecticidal and many others properties positively affecting human health [20]. Walnut is categorized as a strategic species for human nutrition because the food and agriculture organization (FAO) included it in the group of priority plants [21]. Not only are walnuts used in human nutrition but also the young green walnuts are much appreciated in the traditional folk medicine of some countries and the preparation of jams and a wholesome alcoholic drink named walnut liqueur which contains high levels of phenolic compounds and vitamins. The fruit is left to steep in food-grade ethanol. This liqueur is made out of fresh walnuts with green husks just before the hardening of the endocarp [22]. Substantially high amounts of by-products rich in phenolic compounds are produced in agricultural industries, which have gained increasing interest due to their excellent antioxidant properties [23]. Walnut is recognized as one of the agro-crops that produce more waste-heavy materials. It is estimated that ~70% of the fruit weight is shell and husk, low-value waste materials that are rich in different chemicals, mainly phenolic compounds [24].

To the best of our knowledge, this review is the most comprehensive paper highlighting the importance of walnut and the first paper describing the extraction, quantification, and comparison of phenolic content in different parts of the walnut tree and fruit using different employed solvents reported in the scientific literature. The current review paper also tries to discuss the antioxidant and radical scavenging activity of each part of the walnut plant.

## 2. Chemical Classes of Metabolites Detected in the Walnut

It has been reported that the health benefits of walnuts are generally related to their chemical profile [25]. According to the studies by Halvorsen, et al. [26], Kornsteiner, et al. [16], and Mishra, et al. [27], walnuts rank as one of the highest content of antioxidants among all the studied seeds and nuts. Arcan and Yemenicioğlu [28] indicated that walnuts exhibited the highest antioxidant activity, followed by pistachios and hazelnuts. Walnuts are considered a good source of tocopherols and essential fatty acids [29,30]. Linoleic acid is the primary fatty acid, followed by linolenic, palmitic, oleic, and stearic acid [29,31,32,33]. The high concentration of polyunsaturated fatty acids has been revealed to decrease the risk of CVDs by reducing the concentration of low-density lipoprotein (LDL) and enhancing the level of high-density lipoprotein (HDL) [34,35,36,37,38,39,40,41,42,43,44]. Kris-Etherton, et al. [38] argued that ellagic acid and flavonoids in walnut have potential serum cholesterol-modulating effects, and one group of flavonoids has cardioprotective effects. It has been reported that catechin inhibits LDL oxidation and protects lymphoid cells against the cytotoxic effects of oxidized LDL [45]. Anderson, et al. [46] demonstrated that walnut extract containing gallic acid, ellagic acid, and flavonoids suppresses the oxidation of human LDL in vitro. Moreover, walnuts possess other compounds that can be beneficial for promoting human health including polyphenols, tannins, folate [47], dietary fiber, protein, melatonin [48], and sterols [29,49]. It has been reported that oil, tocopherols, and fatty acid concentration can fluctuate considerably among various walnut varieties and environmental conditions [30]. Additionally, it has been shown that walnuts have a high concentration of α-tocopherol, a vitamin E family compound, which exhibits antioxidant properties, principally in the inhibition of lipid oxidation processes [30,50]. Recently, juglone compound or 5-hydroxyl-1,4-naphthoquinone, belonging to the class of naphthoquinones, has received much attention because it shows various biological activities, including excellent antitumoral effects on different human cancer cell lines [51,52]. In this way, pyrogallol is another phenolic compound present in different parts of the walnut and their different properties, such as the interaction with serum albumin has been comprehensively investigated [53].

## 3. Different Parts of the Walnut Tree

### 3.1. Walnut Fruit—Different Parts

**Figure 1:**
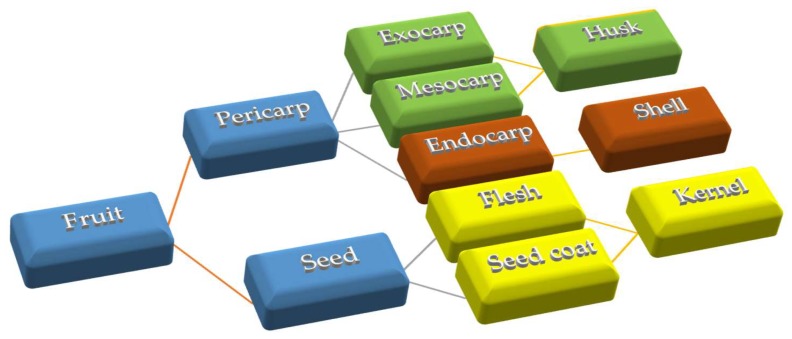
Botanical nomenclature of walnut fruit different parts.

From an anatomical point of view, fruits are composed of a seed and the pericarp (exocarp). The first includes flesh and seed coat, while the second comprises three different parts called exocarp or epicarp (the outer layer of the fruit), mesocarp (the middle part) and endocarp (inside layer). The layers of the pericarp are not distinguishable in dry fruits such as walnut. Thus, it is thought that the husk is both the exocarp and mesocarp, and the shell portion is the endocarp. The enclosed kernel with skin is the walnut seed (Figure 1).

**Figure 2:**
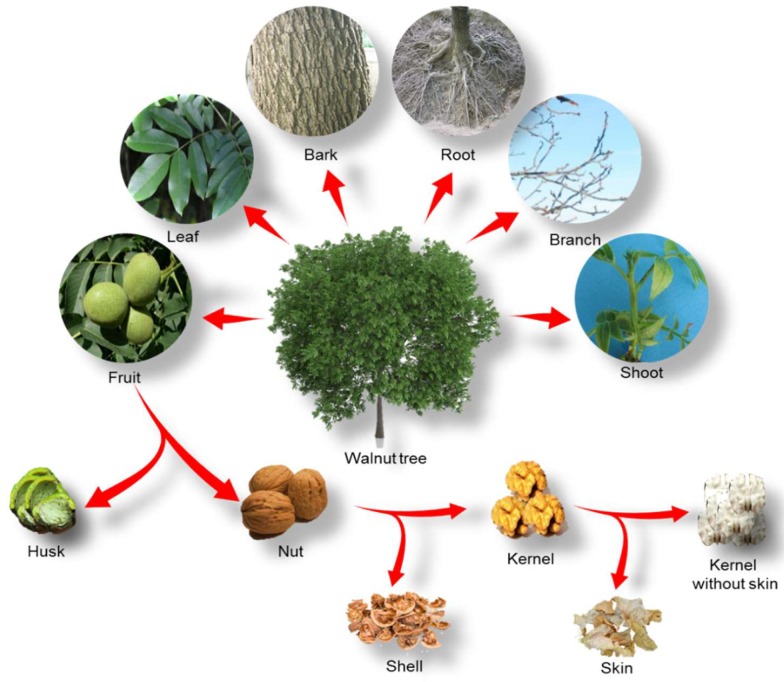
Different parts of the walnut tree and fruit, as illustrated in the schematic image. Fruit, branch, leaf, shoot, and bark are considered as the main parts of the tree which have been comprehensively investigated. Walnut fruit is the most crucial portion of the plant because it contains the kernel. Walnut fruit is composed of five main parts: the outer green husk, the middle part as the shell and, finally, the kernel covered with a brown leathery layer as skin. Inside the nut, the seeds of walnut are separated from each other by a brown woody layer, namely, the pellicle (not shown here).

Undoubtedly, walnut fruit is the most crucial part of the plant for human because it contains the edible kernel. As has been shown in Figure 2, walnut fruit includes five individual parts which are known as kernel, skin, pellicle, shell, and husk. The inner part of the fruit contains the valuable kernel, and it is usually consumed in human nutrition. The kernel is enclosed by a leathery light brown thin layer which is known as skin or seed coat. The high concentration of antioxidants in this part of the fruit protects the kernel from different hazardous effects such as viral, fungal or microbial contaminations and also ultraviolet (UV) irradiation. The shell is the middle portion of walnut fruit that is considered as a hard lignocellulosic material. The kernel is surrounded by the shell which is known as a nut, and it must be mechanically cracked to release the kernel. Inside the nut, the kernel is divided into some parts by the pellicle. The outer part of the walnut fruit is its green husk, which cracks when the fruit on the tree is ready to be harvested and, thus, it can be easily removed [54].

#### 3.1.1. Kernel

Walnut seed is highly nutritious. In Asia and Europe, walnut seeds are also used as a traditional remedy for the treatment of some illnesses such as stomachache and cough [55]. Also, the kernel of *J. regia* L. has been used as antimicrobial, anthelmintic, and antidiarrheal treatment in traditional medicines [56]. The walnut seed or kernel represents 30–40% of the nut weight, depending mainly on the variety. The seed of this tree nut shows high levels of oil (52–70%) in which poly- and monounsaturated fatty acids are predominant [32,57,58,59], as well as oleic acid and linoleic that are sensitive to oxidation. In addition to oil, walnuts provide considerable amounts of proteins (up to 24% of the walnut seed weight), carbohydrates (12–16%), fiber (1.5–2%) and minerals (1.7–2%) [59,60,61,62,63]. Walnuts show a lower concentration of antioxidant α-tocopherol rather than other nuts, such as hazelnuts, almonds, peanuts, etc. [64]. Besides, the lipid autoxidation suppression attributes are connected to the antioxidants present in the walnut seed. Walnut kernel contains high amounts of various phenolic compounds, and the slightly astringent flavor can be related to the presence of phenolic compounds [59,65].

#### 3.1.2. Skin

The flesh of walnut seed is surrounded by a brown leathery coating, called the skin or the seed coat, which protects walnut kernel from oxidation and microbial contamination. Walnut phenolics are measured in the highest concentration in the skin, and they are reported to have favorable effects on human health owing to their apparent anti-atherogenic and antioxidant properties. It has been well evidenced that the removal of the seed coat reduces the total antioxidant activity of hazelnuts, walnuts, and pistachios by approximately 36, 90, and 55%, respectively [28]. The concentrations of three abundant phenolic compounds juglone, syringic, and ellagic acid in walnut seed coat are over 20-fold higher than the concentrations of these compounds in the kernel [65]. It has been reported that the mean of the total phenolic content (TPC) of seed coat was 12.7-fold higher than that of the kernel, but the antioxidant activity (AA) is 12.9-fold higher [66]. Thus, most of the antioxidants are concentrated in the skin portion of the walnut fruit. There is a handful study that analyzed the potency of walnut seed skin as an antioxidant [67]. However, the research mostly extended on the identification of the bioactive compounds and their antioxidant activity in walnut seed [47,68,69,70], husk [22,71,72,73], shell [54,74,75,76], leaves [77,78,79] and shoot [80,81,82]. Even though it has already been demonstrated that individual walnut components have antioxidant potential and the scientific information on the antioxidant properties of walnut skin bioactive compounds is still rather scarce. Therefore, the evaluation of such properties remains a valuable task, especially for discovering and developing new sources of nutraceuticals, functional foods, and natural antioxidants.

#### 3.1.3. Shell

Walnut shell is a chemically inert, hard, biodegradable and nontoxic material generally used as an abrasive. Only the shell portion of the walnut fruit makes up a considerable percentage of the total weight of the nut and has a wide range of applications. As an agricultural by-product of the walnut processing industry, walnut shell is available in abundant quantities. Walnut shell is also advantageous because it is available in a renewable resource form. It is used as an abrasive to blast clean and polish soft metals, stone, fiberglass, plastics, and wood. Walnut shell provides an efficient way to tumble and polish jewelry, gun casings, metal parts, and ink pens. It also serves as an effective filtration media to separate crude oil [83], hazardous materials [84,85,86,87,88] and heavy metals [89,90,91,92,93] from water. Nowadays, walnut shell filtration [94,95] or its biofiltration capacity [96] is used to treat oilfield-produced water, steel mill direct spray, the refinery of wastewater, ethylene plant quench water, ethyl acetate separation, cooling water, copper concentrate decant and also caster water [97,98,99]. In recent years, the preparation of antioxidant and antimicrobial pyroligneous acids from walnut shell has been described in some research [54,100,101].

#### 3.1.4. Husk

The green cover of walnut fruit surrounding the nut is named the husk, and it is an agricultural waste product which has been widely used in folk medicine for treating skin diseases and alleviating pain. However, in the recent years, it has received increasing attention in modern pharmacology mainly due to its antioxidant properties [102]. Walnut green husk is produced in walnut fruit harvesting and processing. Recently, it has been valued as a source of natural compounds with antioxidant and antimicrobial properties [103]. The effective utilization of husk can be a crucial issue because its use as an abundant supply of phytochemicals will highlight the importance of walnut production, as well as propose applications for a by-product, which is produced in a high quantity [73].

### 3.2. Other Parts of the Walnut Tree

Moreover, other portions of the plant such as the leaf, bark, root, shoot, branch, and even the stem or root bark of walnut has also been studied in recent years (Figure 2). Today, the chemical constituents of these parts of the walnut tree were characterized in some research studies, and various potential uses in different fields have been defined.

#### 3.2.1. Leaf

The highly appreciated leaves of walnut contain considerable amounts of phenolic compounds, which are mainly attributed to their excellent pharmacological and therapeutic properties [104]. They are easily available in high quantities, while the other parts of the tree such as bark are not abundant and plant life depends on it. Walnut leaves serve as a source of health care compounds and have been extensively used as remedies in the conventional medicine for the treatment of hemorrhoidal symptomatology, venous insufficiency, and for its anthelmintics, depurative, antidiarrheal and astringent properties [105,106]. Anti-scrofulous, hypotensive, antifungal, keratolytic, hypoglycemic, and sedative activities have also been reported for the extracts derived from walnut leaves [107,108,109,110,111,112]. In Portugal and some other European countries, particularly in the rural areas, dried walnut leaves are often used as an infusion. Juglone is the natural phenolic compound in walnut, which is found in fresh walnut leaves [113,114]. The leaves of *J. regia* L. are considered as a good source of flavonoids [115] and other beneficial compounds [116,117].

#### 3.2.2. Shoot

The shoot is the main bearing structure in the mature walnut tree and is thus important for tree productivity [118]. The phenolic composition of the walnut shoot is critical in its susceptibility to bacterial blight *Xanthomonas campestris* pv. *juglandis* (*X. campestris* pv. *juglandis*), a common disease with the greatest economic importance in all walnut production centers [80]. Cheniany, et al. [81] observed that the rhizogenesis of walnut shoot is associated with its flavonoid content. The earlier investigation has proved the presence of flavanol myricitrin and hydrojuglone β-d-glucopyranoside (HJG) as a glucoside derivative of juglone in the walnut shoot [119]. Radix, et al. [120] and Mahoney, et al. [121] described that juglone is the main phenolic compound in walnut defense mechanism against pathogens while both myricitrin and HJG are known as two main phenolic markers of rejuvenation in walnut trees. Solar, et al. [80] and Cheniany, et al. [82] reported different phenolic acids (*p*-coumaric, gallic, vanillic, syringic, ellagic and chlorogenic acid), flavonoids (quercetin, catechin, and myricetin) and quinones (juglone and 1,4-naphthoquinone) in walnut shoot.

#### 3.2.3. Bark

Walnut bark is known as a resinous and scented material. The dried stem bark can be used as a tooth cleaner. It is also employed as a dyeing agent for staining the lips in some countries [122]. Walnut leaves and bark in the decoction form are used with alum for the brown coloring of wool [123]. It has been claimed that it possesses anthelminthic, diuretic, laxative, astringent, anti-inflammatory, blood purifying, anticancer, depurative, and detergent properties. The antifungal, antibacterial, and antioxidant activities of this part of walnut have been studied [124]. It has been reported that the aqueous extract of walnut bark protects mice against cyclophosphamide-induced biochemical toxicity [125]. It contains several therapeutically active constituents, especially polyphenols [126,127]. *J. regia* L. stem bark contains chemical constituents, namely β-sitosterol, ascorbic acid, juglone, folic acid, gallic acid, regiolone, and quercetin-3-α-l-arabinoside [128].

#### 3.2.4. Root

Scientific information regarding the chemical constituents of walnut root is scarce because this part of the tree is not easily available. Alkaloids, phenols, flavonoids, steroids, terpenoids, tannins, and saponins are the main phytochemicals reported to be present in the walnut root extract [21]. The root part of the walnut tree was also investigated as a potent source for obtaining effective antifungal or antibacterial constituents. The prepared methanolic extracts derived from the walnut root are effective against common uropathogenic bacteria causing urinary tract infections (UTIs) [129] and different types of *Candida* strains [21].

#### 3.2.5. Branch

In recent years and by using the top grafting method, new varieties gradually replaced the poor fruit quality and low yield traditional walnuts. During the renewal procedure, many branches were produced as agro-forest waste. Comprehensive reports are not available in the literature regarding the phenolic composition and the antioxidant activity of walnut branch. The wasted walnut tree branch as a bio-resource was only used to prepare pyroligneous acid using the pyrolysis process, and the antibacterial and antioxidant activities of different chemical constituents of pyroligneous acid were reported [130].

## 4. The Solvents Used for the Preparation of Walnut Extracts

Water, ethanol, methanol, ethyl acetate, acetone, hexane, dichloromethane, petroleum ether, chloroform, *n*-butanol, benzene, and cyclohexane are the common solvents which have been used for the preparation of extracts from different parts of the walnut fruit and tree (for more details, see Table 1). Among them, water, ethanol, methanol, ethyl acetate, and acetone are the most used extraction solvents. There is some research in the literature explaining the appropriateness of the considered solvents mentioned above and their selection in most investigations worked on walnut. For example, the phenolic content and antioxidant activities of methanolic and petroleum ether extracts derived from the seed, husk, and leaf parts of the walnut were investigated [131]. The highest levels of phenolic content and correspondingly antiradical activities were seen in seeds, leaves and green husk methanolic extracts, respectively. In contrast, petroleum ether (a mixture of some alkanes, e.g., hexane, heptane, and pentane) has no or less potential for antioxidant activity. The results of some other studies demonstrated that in most cases, the obtained extracts from different parts of walnut using alcohols, water or a mixture of them presented a higher phenolic content than the extracts of other nonpolar solvents such as petroleum ether, chloroform, n-butanol, benzene, and cyclohexane. It is believed that polar solvents such as methanol, ethanol, water, acetone, and ethyl acetate are the best solvents for phenolic extraction.

### 4.1. Fruit

#### 4.1.1. Kernel

Among different considered solvents, water, ethanol, methanol, and acetone are the most used solvents employed for the preparation of phenolic extracts from walnut kernel (refer to Table 1 for further details). It has been shown in some studies that among the solvents used, methanol is considered as an appropriate solvent for the extraction of phenolics. The prepared methanolic extracts possess higher antioxidant content and radical scavenging activity. For example, Carvalho, et al. [131] prepared methanolic, and petroleum ether extracts from walnut seed, green husk, and leaf and evaluated their TPC and antioxidant properties. It has been demonstrated the highest TPC, and DPPH scavenging activity for the methanolic seed extract, followed by leaf and green husk and much lower or absent antioxidant action was seen for the prepared petroleum ether extracts. In an investigation to evaluate the antioxidant activity, phenolic, and mineral contents of the walnut kernel as a function of pellicle color among different genotypes, authors used methanol as the extraction solvent [66]. Kafkas, et al. [132] characterized the fatty acid profile, tocopherols and total phenol content of 10 walnut cultivars, namely Bilecik, Chandler, Hartley, Howard, Maraş 12, Maraş 18, Midland, Pedro, Şen and Serr. The methanolic extracts were used for the determination of the TPC of kernel samples from different selected walnut cultivars. In another study by Dodevska, et al. [133], the quality of fifteen selected Serbian plant foods was characterized, and the methanolic extracts were considered for assessing the TPC of walnuts. It has been shown that the methanolic extract of walnut and the component ellagic acid in the extract exhibit anti-inflammatory activity in human aorta endothelial cells and osteoblastic activity in the cell line KS483 [134]. The results of another study using the prepared methanolic extract demonstrated that walnut modulates the activation of microglia through alteration in intracellular calcium concentration [135].

The high safety of water makes it a suitable solvent which was used in some investigations as the extraction solvent for the preparation of extract from the walnut kernel and the evaluation of its antioxidant properties. In an investigation carried out by Pereira, et al. [69], the chemical composition, antioxidant potential and antimicrobial activity of six walnut cultivars (Franquette, Lara, Marbot, Mayette, Mellanaise, and Parisienne) cultivated in Portugal were considered. In this work, boiling water was used for the preparation of extracts. Moreover, the artificial neural network (ANN) and multiple linear regression were used for the prediction of the antimicrobial activity of the walnut kernel and the extracts were prepared using water as the extraction solvent [136].

In the case of ethanol as the employed extraction solvent, the aqueous and ethanolic extract of walnut seed were used to assess antioxidant content and radical scavenging activities. The ethanolic extract showed significantly higher antioxidant activity in comparison to the aqueous extract [137]. In contrast, Arcan and Yemenicioğlu [28] found that the ethanolic extracts of walnut kernel contained lower amounts of phenolic compounds than the aqueous extracts obtained before ethanol extraction. They considered the antioxidant activity and phenolic content of fresh and dry hazelnuts, walnuts and pistachios with or without the seed coat and observed some variation in phenolic content and antioxidant activity for hydrophilic and ethanolic fractions obtained by successive extraction of the walnut kernel.

It has been reported that the prepared extract using acetone had higher antioxidant activity than when using water and even methanol. In this investigation, the effect of processing techniques (microwaving and soaking) on walnut antioxidant and antiproliferative activities was evaluated [138]. The extracts were obtained by using different solvents (acetone, methanol, and water) and compared to the methanol and water extracts; acetone extract showed considerably higher antioxidant activity. The antioxidant activity was increased after soaking treatment and, thus, the authors claimed that the soaking process could be utilized for promoting the health benefits of walnuts in industrial applications.

It has been shown that a mixture of different solvents and water can be used as an effective extraction system for the evaluation of antioxidant and antiradical activity of walnut seed. As a result, a mixture of methanol and water with a different ratio has been reported. For example, high-speed counter-current chromatography (HSCCC) and electrospray ionization ion-trap time-of-flight mass spectrometry (ESI-IT-TOF-MS) methods were combined and established for preparative isolation of complex mixtures of compounds from polar extracts of walnut. In this study, the authors used methanol 80% for the preparation of walnut kernel crude extract [179]. A mixture of methanol and water was also employed for the assessment of individual phenolic compounds, TPC, and antioxidant potential in kernels, oils, and bagasse pellets of different walnut cultivars [180]. Eight genotypes of Turkish walnuts were selected by Cerİt, et al. [181] to assess their kernels from the viewpoint of glutathione and TPC, as well as their antioxidant capacities. In this work, the authors used methanol:water (70:30) solution for the preparation of extracts. A mixture of methanol and water with the same ratio was also reported by Rosales-Martínez, et al. [178] when they compared the antioxidant activities of phenolic extracts from Mexican peanuts, peanuts’ skins, nuts, and pistachios. The methanolic extracts with the same ratio were also used for the evaluation of eleven different kinds of nuts, including cashew nut (*A. occidentale* L.), pistachio (*P. vera* L.), pinhao seed (*A. angustifolia* (Bertol.) Kuntze), hazelnut (*C. avellana* L.), peanut (*A. hypogaea* L.), chestnut (*C. sativa* Mill), walnut, pecan (*C. illinoinensis* (Wangenh.) K. Koch), Brazil nut (*B. excelsa* Humb. & Bonpl.), macadamia (*M. integrifolia* Maiden and Betche), and almond (*P. dulcis* (Mill.) D.A. Webb) [176].

A mixture of ethanol and water was examined in some research as an effective extraction solvent system. Authors used a different ratio of ethanol and water for enhancing extraction efficiency. Gómez-Caravaca, et al. [155] aimed to develop a capillary electrophoresis–mass spectrometry (CE-MS) method for the identification and quantification of phenolic and other related polar compounds in the walnut kernel. Using rapid analytical techniques, Verardo, et al. [156] analyzed the lipid fraction and characterized phenolic compounds in Italian walnuts (Chandler cv.) grown in the same experimental orchard under different agronomical conditions. In both types of research, phenolic extracts were prepared using a mixture of ethanol and water. Additionally, it has been observed that pancreatic lipase activity was inhibited by 50% ethanolic extract of walnut seed in vitro, and it showed the hypolipidemic effect on high fat diet-induced obese Mice [157]. To evaluate the effect of skin removal on the solubility of protein fractions from walnut flour, Labuckas, et al. [154] extracted the phenolic fraction from the skin and walnut flour and examined their antioxidant capacity. In this investigation, two different solvent systems (a mixture of ethanol and methanol with water) were employed, and minor differences were observed among the solvent systems used concerning TPC and antioxidant activity.

In most studies dealing with the antioxidant content and radical scavenging activity of walnut seed, diluted acetone with water was also used as an extraction solvent. Christopoulos and Tsantili [199] investigated the effects of cultivar and some storage conditions such as time, temperature, and oxygen availability on the content of total phenolics, the total antioxidant capacity and the color of walnut kernels from three different cultivars including Chandler, Hartley, and Ioli. The authors used 80% acetone as the extraction solvent for obtaining phenolic extracts. Anderson, et al. [46] prepared walnut kernel extract using 75% acetone and reported that the oxidation of human plasma and LDL is inhibited in vitro by walnut polyphenols. Using the acetone extract of walnut kernel demonstrated that polyphenol extract attenuated the immunotoxicity induced by 4-pentylphenol and 3-methyl-4-nitrophenol in Murine splenic lymphocyte [202]. A mixture of acetone and water was also reported in the scientific reports for the comprehensive identification of walnut polyphenols by the liquid chromatography coupled with electrospray ionization hybrid linear trap quadrupole-Orbitrap (LC–LTQ-Orbitrap) mass spectrometry. One hundred and twenty different compounds, belonging to the class of hydrolyzable and condensed tannins, flavonoids, and phenolic acids were identified in this investigation [200]. By using 80% acetone, the antioxidant and antiproliferative activities of almonds, Brazil nuts, cashews, hazelnuts, macadamia nuts, pecans, pine nuts, pistachios, and walnuts as commonly available tree nuts and peanuts in the United States were evaluated [25].

The use of other solvents for the preparation of walnut kernel extract is limited to n-butanol or ethyl acetate. In an investigation by Zhang, et al. [70], the authors used an activity-directed fractionation and purification process in order to isolate DPPH radical scavenging components from walnut kernels. In their study, ethyl acetate and n-butanol fractions represent higher DPPH radical scavenging activities compared to those fractions of petroleum ether and water. Glansrins A, B, and C as three hydrolyzable tannins along with adenosine, adenine, and 13 other tannins were characterized in the n-butanol extract of walnut seeds [68].

#### 4.1.2. Shell

It has been indicated that the extracted phenolics from the walnut shell can be used as a potential source of natural antioxidants for application in the food and pharmaceutical industries. However, the research on the extraction and analysis of the walnut shell chemical composition are not fully considered, and the research is limited to several studies. In a recent study, the extraction of phenolic compounds from the walnut shell using the ultrasonic bath, ultrasonic probe, and standard shaking method was reported. For all three extraction methods, a mixture of 50% ethanol/water was used as a solvent. The achieved extraction yield by an ultrasonic probe was 51.2 mg gallic acid equivalents/g dry weight (GAEs/g DW), 2-fold higher than both shaking and ultrasonic bath methods which were 20.6 and 25.8 mg GAEs/g DW, respectively. By a size reduction of the walnut shell, the extraction of phenolic compounds was more improved. When the particle size was between 45–100 mesh, the best extraction yield of 52.8 mg GAEs/g DW was attained. The authors found that the ultrasonic probe treatment is the best method for the extraction of phenolics from the walnut shell. The scanning electron microscopy (SEM) images indicated that during the ultrasonic probe treatment, the solid structure of the cells was better ruptured increasing the penetration of solvents and, thus, enhancing the extraction yield [24]. In another study, Soxhlet extraction (SE) followed by pretreatment with microwave irradiation (MWI) and ultra-sonication using two different solvents (methanol and acetone) was optimized by a three-factor three-stage response surface methodology (RSM) based on the central composite design (CCD) [183]. The obtained extract was optimized using RSM. They observed that pretreatment enhanced the total extract yield compared to conventional SE. The authors reported that pretreatment with MWI was more effective than ultra-sonication. Besides, as a solvent, the performance of acetone was better than methanol in the bio-components recovery. In comparison to the conventional SE, MWI pretreatment enhanced separation for methanol and acetone by 4.06- and 5.25-fold, respectively. The highest yield (46 mg/g) was obtained when the sample was pretreated with MWI at 180 W for 4 min using acetone as the solvent. The extraction yields, total flavonoids content (TFC) and antioxidant properties of the walnut shell using different solvents (water, chloroform, methanol, ethanol, ethyl acetate, and n-butanol) were analyzed, and authors achieved the highest extraction yield with n-butanol (4.54%) [140,141].

#### 4.1.3. Husk

Water, methanol, and ethanol were recognized as the most suitable solvents for the preparation of an extract from the walnut husk (see Table 1 for more details). A study on the effects of different extraction solvents such as hexane, ethyl acetate, acetone, ethanol, methanol, and water on the content of phytochemicals showed that the extraction solvents significantly affected the phytochemical content and antioxidant activities of the extracts obtained from walnut green husk. It has been reported that acetone, ethanol, and methanol extracts had higher contents of phytochemicals, and exhibited stronger antioxidant activities, followed by the ethyl acetate and water extracts, and the lowest for the hexane extract [18].

Among the employed solvents, methanol is the best solvent considered as the first choice by some authors in the preparation of walnut husk extracts. The antioxidant activity of the endocarp and exocarp of walnut were evaluated using different solvents such as dichloromethane, ethyl acetate, methanol, and water. The highest antioxidant activity was achieved for the methanol extract of walnut exocarp [56]. In an investigation reported by Akbari, et al. [67], the phenolic compounds composition, antioxidant content, and antiradical activity of six different genotypes of walnut fruits grown in Iran were studied. Different parts of the collected fruit samples, including kernel, skin, shell, and husk were extracted with methanol using Soxhlet apparatus. In another similar study, the husk and kernel of seven different walnut genotypes from Iran including KZ7, KZ9, KZ15, OR126, Sebin, Pedro and Chandler were extracted using methanol in Soxhlet apparatus [182]. The phenolic response in green walnut husk after infection with bacteria *X. arboricola* pv. *Juglandis* has also been investigated by the preparation of methanolic extracts [184]. The research was performed on healthy green walnut fruits and infected fruits. Mishra, et al. [27] investigated the antioxidant activity of some dry fruits (almonds, walnut, cashew nut, raisins, chironji) through methanolic extracts. To assess the influence of environmental factors on antioxidant activity, the phenol and flavonoids content of walnut husk from 11 regions of Iran with different geographical and climatic conditions, a methanolic extract was prepared and used [71].

Ethanol is also a useful solvent for the effective extraction of phenolic from the walnut husk. The phytochemical screening and the high-performance thin layer chromatography fingerprinting (HPTLCFP) analysis of walnut green husk using different solvents (water, ethanol, chloroform, petroleum ether, and ethyl acetate) was investigated, and the high extraction yield was achieved by ethanol (7.12%) [142].

In some research studies, water was directly used as the extraction solvent. The green husks of five different walnut cultivars including Franquette, Mayette, Marbot, Mellanaise, and Parisienne were considered for the obtainment of aqueous extracts, and their TPC, antioxidant and antimicrobial activities were studied. Walnut green husk was extracted with boiling water [73]. Recently, Izadiyan, et al. [143] tried to establish a green chemistry technique for the environmentally friendly synthesis of gold nanoparticles (Au-NPs). In this research, *J. regia* L. green husk extract was prepared using boiling water for 30 min and, then, it was successfully used as the stabilizer and reducing agents for the biosynthesis of NPs. They claimed that when using walnut husk extract as a natural plant resource, NPs with the preferred size could be obtained.

The results of some studies revealed that the selection of an appropriate ratio of different solvents and water could increase the content of phenolics in the prepared extracts derived from walnut green husk. Most research studies used a mixture of ethanol, methanol, or acetone as an effective extraction system. For example, Fernández-Agulló, et al. [103] tried to analyze the effect of different solvents (water, methanol, ethanol and 50% aqueous solutions of methanol and ethanol) on extraction yields and extract bioactive properties of walnut green husk. They obtained the highest extraction yield with water (44.11%), and high bioactive potential has been achieved by the samples extracted with water/ethanol (1:1) (84.46 mg GAEs/g extract; EC_50_ = 0.95 mg/mL for reducing power (RP) and EC_50_ = 0.33 mg/mL for DPPH assay). In another investigation, a comparison between the conventional and ultrasonic-assisted extraction (UAE) of natural antioxidants from walnut green husk has been reported in studies reported by Tabaraki and Rastgoo [161]. They applied ultrasonic technology for the extraction of antioxidants from walnut green husk, and ethanol was used as a food-grade solvent. A mixture of 60% ethanol–water mixture as a solvent, a temperature of 60 °C, and an extraction time of 30 min were reported as the optimal conditions. Authors compared UAE and conventional extraction and showed that TPC, ferric-reducing antioxidant power (FRAP), DPPH and the extraction yield obtained by UAE during 30 min were significantly higher than by the conventional extraction during 16 h. Different extracts were prepared from green fruits of ‘Sibisel 44’ walnut cultivar and, then, their TPC and TFC, antioxidant activity, individual phenolics content, and color component were investigated [160]. In this study, the authors reported that for the TPC and TFC extraction from green walnut fruits, 70% ethanol was more efficient.

In the study of Boncler, et al. [201], the authors used a mixture of acetone and water for the preparation of phenolic extracts from walnut green husk and the spent hops (*H. lupulus* L.). In this investigation, the prepared extracts were used to assess the anti-proliferative effects of plant extracts on human endothelial cells and compare the PrestoBlue and MTT assays in cellular viability. The cytotoxic and anti-platelet activities of polyphenolic extracts from *A. montana* flowers and *J. regia* L. husks have been compared and for the preparation of both plant extracts, authors used the acetone–water solution as the extraction solvent system [203]. The high content of polyphenolic compounds and antioxidant properties has been described for the prepared aqueous acetone extract of the husk when its antiplatelet aggregation activity was evaluated [102].

### 4.2. Other Parts of the Tree

#### 4.2.1. Leaf

It is clear from Table 1 that water, ethanol, and methanol are considered as the most appropriate solvents for the extraction of phenolic constituents from walnut leaves. Also, some detailed studies available in the literature demonstrate the effectiveness of such solvents. In an evaluation, the anti-proliferative, cytotoxicity and antioxidant activity of walnut leaf extract using different solvents (water, methanol, ethanol, acetone, and benzene) revealed that the methanolic and aqueous extracts might be subjected to further experiments because they contain several secondary metabolites, mainly phenolic compounds [145].

Methanol is also reported as a suitable solvent for the preparation of walnut leaf phenolic extract. The antifungal activity of four prepared extract fractions from the leaves of *J. regia* L. including methanolic, ethyl acetate, alkaloid and hydrolyzed methanolic were evaluated against pathogenic *Candida albicans* strains. The methanolic extract of the walnut leaf was reported as having the highest anticandidal activity followed by the alkaloid, ethyl acetate, and hydrolyzed methanolic extracts [192]. A full investigation was performed on the chemical analysis of Tunisian *J. regia* L. stem and leaf fractions, their antibacterial potential, and dyeing properties. Both water and pure methanol were employed as a solvent for the extract preparation from the stem and leaves of walnut to assess the effect of the solvent on the extraction yield. The obtained extraction yields were 21.45 and 19.37%, respectively, for stem and leaves using methanol as the solvent. However, the values of the extraction yields in the case of water were 12.75 and 15.22%, respectively, for the stem and leaves. From the registered results, the authors reported that methanol is a suitable solvent, and it is kept back for further assays [148]. In another study, phytochemicals and nutraceuticals of 50 selected walnut genotypes were assessed using their methanolic leaf extracts [193]. It has been highlighted in the studies of Khan, et al. [194] that the obtained crude methanolic extract of walnut leaf possessed high anti-pseudomonal activity. Nour, et al. [188] reported the determination of phenolic acids, juglone, and flavonoids in the prepared methanolic extracts of walnut leaves by high-performance liquid chromatography (HPLC). Santos, et al. [189] investigated the leaves and decoction of *J. regia* L. and reported that the methanol extract had higher antioxidant and antitumor potential than a decoction. The antioxidant activity of methanolic and water extracts obtained from nine different fruit tree leaves including avocado, walnut, mulberry, fig, carob, lemon, pomegranate, grape, and loquat collected from the Mediterranean region of Turkey was investigated and it has been found that the methanolic extract had higher antioxidant content and radical scavenging activity compared to aqueous extract [146].

Ethanol is another solvent used in some investigations for the preparation of walnut leaf extracts. According to the study of Einali, et al. [149], they evaluated the antioxidant and antimicrobial activities of ethanol and water extracts of *P. aphylla*, Persian walnut, and oleander leaves. The ethanol extracts with the highest amount of total phenolics and flavonoids in all assays showed the highest antioxidant and antimicrobial activities compared to water extracts. A randomized, double-blind, placebo-controlled clinical trial was also designed by Hosseini, et al. [110] to study the effects of the prepared ethanolic extract obtained from *J. regia* L. leaf on hyperglycemia and lipid profiles in type 2 diabetic patients. The cyclohexane and ethanol extract of the walnut leaf was also used for investigating its effect on the activity of sorbitol dehydrogenase enzyme in diabetic male rats [171]. Nour, et al. [104] tried to establish the optimum conditions for extracting phenolic compounds using the UAE and RSM from walnut leaves. It has been indicated that the liquid-to-solid ratio and ethanol concentration were proved to be the primary factors affecting extraction efficiency. The ethanolic extract of walnut leaves was also examined for antibacterial activities on *Propionibacterium acne* [166].

Distilled, acidified or boiling water was also selected as another solvent for the extraction of antioxidant phenolic compounds from the leaves of walnut. In a first human trial conducted by Hosseini, et al. [109], the aqueous extract of walnut leaves was investigated for its hypoglycemic effect in diabetic patients. Eser, et al. [147] evaluated the effect of acid pre-treatment on the dyeing performance of walnut leaves on wool fibers. They prepared the extract with distilled water using a Soxhlet type apparatus at its boiling point. In another investigation by Amaral, et al. [144], to assess the effects of cultivar, geographical location, and crop season influence on the phenolic profile of walnut leaves, acidified water was used for the preparation of extracts. Recently, the extraction of phenolic compounds from walnut leaves using heat-assisted extraction (HAE) and deep eutectic solvents based on carboxylic acids and choline chloride has been established [150]. The authors performed a preliminary solvent screening using a selected group of carboxylic acids as hydrogen bond donors. The extract of phenolic compounds with the highest yield was obtained using choline chloride mixtures with butyric or phenyl propionic acid at a mole ratio of 1:2, with 20% of water.

In the case of walnut leaves, a mixture of various organic solvents and water as a useful extraction system for obtaining and analyzing the antioxidant content of the prepared extracts has been considered. In this way, different ratios of water and other solvents were employed by different authors. For example, the microwave-assisted extraction (MAE) method was compared with three others for the extraction and determination of TPC and TFC in walnut leaves. It was found that TPC and TFC for the prepared extracts of walnut leaves in MAE method was 4 min, higher than that when using maceration extraction (ME), UAE, and SE for 24 h, 15 min, and 6 h, respectively. Compared with other extraction methods, MAE reduced the extraction time. It was found that blanching had no significant effect on the TPC of walnut leaves. Different methanol concentrations (20, 40, 60, 80, 99.8% *v*/*v*) were tested and the highest TPC was obtained using 60% methanol [187]. The hydroethanolic leaf extract of walnut (20–70% *v*/*v*) was prepared to evaluate their antidiarrheal and antinociceptive effects in rats [170]. The effect of 50% ethanolic extract prepared from walnut green husk and leaf on cutaneous Leishmaniasis caused by Leishmania major in BALB/c mice were also reported by Sharafi Chegeni, et al. [162]. In a double-blind, placebo-controlled clinical trial study reported by Rabiei, et al. [173], a hydroalcoholic extract (70%) of walnut leaves was used to evaluate its effects on blood glucose levels in type 2 diabetic patients and the major cardiovascular risk factors.

In an investigation by Vieira, et al. [172], they tried to compare the ME and MAE methods for enhancing the extraction of valuable compounds from walnut leaves. For optimizing time, temperature and ethanol–water proportion as the main variables of the ME and MAE systems, an experimental design assisted by RSM was developed. The achieved optimum conditions have been reported as 112.5 min, 61.3 °C and 50.4% of ethanol for the ME method; and 3.0 min, 107.5 °C and 67.9% of ethanol for MAE method. Authors reported the highest TPC and total flavonol content (TFOC) for the MAE method. The hydroethanolic leaf extract was obtained by HAE (50.4%, *v*/*v*; 30 g/mL at 61.3 °C for 116 min) at the same optimized conditions for the analysis of phenolic compounds [206]. For the qualitative and quantitative analysis of flavonoids from *J. regia* L. leaves using high-performance liquid chromatography coupled tandem mass spectrometry with electrospray ionization and negative ion detection (HPLC-ESI-MS/MS), Zhao, et al. [167] used 60% ethanolic solution as the extraction solvent. The effect of walnut leaf extract on cerebrum malformation in the fetuses of diabetic rats was studied using ethanol as the extraction solvent. In these studies, the powdered leaves were then allowed to soak in 70% ethanol and, at room temperature, re-extracted three times with fresh 96% ethanol [168,169]. The ethanol extract (80%) of Iranian walnut leaves was examined for its antibacterial activities on *Streptococcus mutans (S. mutans), Streptococcus salivarius (S. salivarius), Streptococcus sanguis (S. sanguinis), and Actinomyces viscosus (A. viscosus)* using the microdilution method. It has been reported that Iranian walnut leaves had antibacterial effects on the examined bacteria and may be a suitable alternative remedy for the protection and treatment of dental plaque due to these microorganisms [164]. Salimi, et al. [165] tried to assess the cytotoxicity effects of various *J. regia* L. leaf extracts in human cancer cell lines. They prepared total extracts with ethanol:water (80:20) at room temperature. Also, the leaf powder was extracted sequentially by solvents with different polarities, including hexane, chloroform, ethyl acetate, and methanol using the ME method. They observed that the yield of extraction for aqueous ethanol was 21.66%, while the yields for the obtained fractions of hexane, chloroform, ethyl acetate, and methanol were 8.66, 2.13, 2.68 and 12.01%, respectively.

In the case of other solvents, it has been shown that the prepared hexane extract of the walnut leaf using hot extraction inhibited the growth of human prostate cancer cells by inducing the apoptosis [205]. The constituents of the chloroform extract of *J. regia* L. leaves were characterized and, then, the anti-proliferative and apoptotic activities were studied [79]. The authors pulverized and mixed the dried leaves with hexane using the percolation method, for 24 h. After the removal of hexane, the obtained powder was suspended and, then, extracted with chloroform.

#### 4.2.2. Shoot

As has been shown in Table 1, methanol as a suitable solvent was also used for the preparation of walnut shoot phenolic extracts. In the first report, in order to identify the potential involvement of phenolics in the constitutive chemical resistance of the shoots against walnut bacterial blight (*X. campestris* pv. *juglandis*), quantitative analyses of different phenolics in annual shoots were conducted [80]. During the growing season, authors measured the content of eight phenolic compounds in the prepared methanolic extracts from rejuvenated annual shoots of walnut cultivars Elit, Franquette, Hartley, and Sampion by HPLC with the photodiode array (PDA) detector. In the second study, Cheniany, et al. [82] considered the content of different groups of phenolic compounds present in the micro shoots of *J. regia* L. cultivars and studied their antioxidant activity using 80% methanol as the extraction solvent. Finally, the same percentage of this solvent was used for the preparation of extracts from micro shoots of *J. regia* L. expressing chalcone synthase. This enzyme influences the content of flavonoid and the frequency of the rhizogenesis of micro shoots [81].

#### 4.2.3. Bark

Walnut bark is also considered as another rich source of phenolic antioxidants. For this reason, different solvents for the effective extraction of phenolic compounds from walnut bark have been examined. Hasan, et al. [196] prepared organic extracts using methanol, chloroform and n-hexane as solvents from walnut root bark and evaluated their effect on cell proliferation, and the molecular mechanism of cell death induction by studying the expression of Bcl-2, Bax, caspases, Tp53, Mdm-2 and TNF-α in MDA-MB-231 human breast cancer cells. It has been proved that the hydroalcoholic extract of the stem bark of *J. regia* L. had a good effect against methicillin-resistant *Staphylococcus aureus (S. aureus)* and the authors recommended that the obtained extract can be used as a commercial disinfectant in the form of spray or solution in hospitals and public places [174]. Additionally, water, methanol, ethyl acetate, and diluted acetone extracts of natural and colored *J. regia* bark were screened for in vitro activity against vaginal *Candida* strains [152]. Authors reported that among four *J. regia* L. extracts for natural and colored bark, the methanolic extract of natural bark had the best antifungal activity against all vaginal *Candida* strains. Ethanolic and aqueous extracts of Iranian *J. regia* bark were prepared. Then, the effects were evaluated on four different oral bacteria, *S. mutans*, *S. salivarius*, *S. sanguis*, and *S. aureus* [128]. In this investigation, the authors concluded that the ethanolic extract of walnut bark had a significant antibacterial effect against all the tested bacteria. It has been shown that the aqueous extract of walnut bark had a significantly inhibited the growth of oral microbial flora compared to the acetone extract [153].

Two samples of *J. regia* tree bark collected from two different regions were extracted using methanol. Evaluation of the antimicrobial properties showed that both extracts possessed significant activities against almost all the tested pathogenic microorganisms [197].

The antibacterial, anticandidal, and antioxidant activities of walnut bark extract were studied with a mixture of acetone:water (80:20; *v*/*v*), ethyl acetate and methanol [195]. This investigation showed that the ethyl acetate extract of walnut bark was more effective against Gram-positive and Gram-negative bacteria and the different species of *Candida*. Methanol, ethyl acetate, and diluted acetone extracts from the fresh and dry stem of *S. persica* and walnut bark were tested for their potent antifungal activities against some *Candida* species. It has been shown that among the prepared extracts of *S. persica* and *J. regia* L., the ethyl acetate extract of walnut bark had the potent antifungal activity against all the studied of *Candida* strains [124].

In the ascending order of polarity, walnut stem bark was extracted using different solvents, including petroleum ether, benzene, chloroform, acetone, methanol, ethanol, and distilled water. The methanolic extract showed significant activity against *Aspergillus niger (A. niger)*. Acetone extract significantly inhibited the growth of *Alternaria alternata* (*A. alternata*), and chloroform extract exhibited promising activity against *Trichoderma* virens (*T. virens*) and *Fusarium solani* (*F. solani*) [151].

#### 4.2.4. Root

The antifungal effect of the ethanolic root extract of walnut was reported in the literature, and it has been examined against nine different strains of *Candida* [21]. The inhibitory activities of walnut root extracts on different isolates of bacteria including *Escherichia coli* (*E. coli*), *S. aureus*, *Staphylococcus saprophyticus* (G^−^ bacteria), and *Klebsiella pneumonia*, *Proteus mirabilis* and *Pseudomonas aeruginosa* (G^+^ bacteria) compared to the control were assessed. Among the three different root extracts tested against six pathogenic bacteria, the ethyl acetate extract was effective against all pathogenic bacteria with the highest inhibition, followed by the methanolic extract while the hexane extract was also effective against relevant agents with moderate inhibition zones [129].

#### 4.2.5. Branch

By executing the pyrolysis of walnut tree branches at three temperature ranges, 90–230, 230–370 and 370–450 °C, pyroligneous acid was produced. A total of 63.46% of the total constituents of the produced pyroligneous acid were phenols and organic acids. The collected pyroligneous acid at 230–370 °C showed most potent antimicrobial activity against *S. aureus*, *E. coli*, *Bacterium proteus* (*B. proteus*), *Bacterium prodigious* (*B. prodigious*), *Aerobacter aerogenes* (*A. aerogenes*). Moreover, all the pyroligneous acids exhibited inhibition activities on the tested plant pathogens: *Phytophthora capsici* (*P. capsici*), *Colletotrichum orbiculare* (*C. orbiculare*), *Valsa mali* (*V. mali*), *Cochliobolus sativus* (*C. sativus*), *Helminthosporium sativum* (*H. sativum*), and *Phytophthora infestans* (*P. infestans*). The maximum inhibition percentage (100%) indicates that the obtained pyroligneous acids at higher temperatures present reasonable inhibition activities on plant pathogens [130].

## 5. Total Antioxidant Content

### 5.1. Fruit

The values of TPC, TFC, TFOC, total flavanol content (TFAC), total hydrolyzable tannin content (THTC), and total condensed tannin content (TCTC) for different parts of walnut fruit reported in the literature are summarized in Table 2. From the table, it can be easily observed that in most studies, the contents of total phenolics and flavonoids for different parts of walnut fruit have been evaluated. However, comprehensive data regarding the TFOC, TFAC, THTC, and TCTC are rare.

#### 5.1.1. Kernel

The results of different studies indicate that walnut seed contains higher antioxidant content. Among different considered dry fruits, the methanolic extract of walnut showed a higher value of the antioxidant activity. A higher TPC was reported in walnuts followed by almonds, cashew nut, chironji, and least phenolic content was found in raisins [27]. The antioxidant content (TPC and TFC) and antiproliferative activities of nine different types of tree nuts and peanuts which are consumed in the United States were comprehensively evaluated [25], and walnuts exhibited the highest TPC, TFC, and TAA. Additionally, walnuts and pecans exhibited the highest antiproliferative activity against the proliferation of HepG2 and Caco-2 cells. The chemical analysis and the identification of the bioactive compounds of peanut, nut and pistachio varieties showed that walnut had the highest TPC (1404 ± 23 mg GAEs/100 g) and antioxidant capacity (191 ± 4.2 μmol TEs/g) [178]. The LC-LTQ-Orbitrap technique was applied for the identification of walnut polyphenols, and 120 various compounds belonging to the groups of hydrolyzable and condensed tannins, flavonoids, and phenolic acids were reported [200]. The authors obtained high TPC for walnut seed extract. Abe, et al. [176] registered the highest TPCs and DPPH radical scavenging capacities among eleven different types of nuts, including cashew nut, pistachio, pinhao seed, hazelnut, peanuts, chestnut, walnut, pecan, Brazil nut, macadamia, and almond.

The content of antioxidants may fluctuate among different genotypes and cultivars. The proximate and mineral composition, total polyphenol, fatty acid profile, serotonin and melatonin contents were assessed in four walnut cultivars (cv. Serr, Hartley, Chandler, and Howard) and the highest TPC was registered for Howard cultivar [208]. Akbari, et al. [67] studied the TPC and TFC of different parts of fruits among six genotypes of walnuts grown in Iran. The methanolic extracts of walnut genotypes were analyzed for their RP, DPPH, superoxide anion, and nitric oxide radical scavenging capacity. Remarkable differences in TPC and radical scavenging potential in different portions of fruits and among various considered genotypes were found.

As discussed, there are numerous investigations suggesting that walnuts possess potent antioxidant activity in comparison to the other nuts [16,26,27,210,211] and this observation is probably related to the presence of phenolic compounds [68], particularly hydrolysable tannins [46], tocopherols [16] and also melatonin, an indoleamine that shows high antioxidant activity [48]. Most of these investigations have dealt directly with extracts from the non-defatted raw material, even though several of them have considered the antioxidant capacity of lipophilic compounds of walnut or walnut oil [212]. However, the influence of the two main fractions of walnut (defatted material and oil) on its full antioxidant capacity is not well recognized. In a comprehensive investigation, Arranz, et al. [207] assessed the antioxidant capacity of oil, defatted material, and whole walnut. High TPC and antioxidant capacity for aqueous–organic extracts of the whole walnut were observed by the authors (2016 ± 178 mg GAEs/100 g of dry matter (DM) and 165.18 ± 19.3 µmol Trolox equivalents (TEs)/g of DM by 2,2′-azinobis(3-ethylbenzothiazoline)-6-sulfonic acid (ABTS) assay, respectively). The obtained values confirmed the TPC obtained by Kornsteiner, et al. [16], who measured that the extractable TPC was in the range of 1020–2052 mg GAEs/100 g of DM after the extraction of the whole walnut using a solution of 75% acetone and 25% of 526 µmol/L sodium metabisulphite. An antioxidant capacity of 119.91 µmol TEs/g of DM by ABTS assay for walnut extracts was also reported [210]. They measured the TPC in the aqueous–organic extracts of the defatted matter of walnut. After the defatting sample by petroleum ether, the TPC in the extracts, and the antioxidant capacity were 1071 ± 35 mg GAEs/100 g of DM, and 211.85 ± 9.51 µmol TEs/g of DM by ABTS assay, respectively. The authors concluded that the high-fat content of walnut (66%) affected the values of antioxidant capacity and the TPC of the extracts, an important point that has not been reported in the literature dealing with nuts. Moreover, in extraction residue, the high-fat content formed an emulsion preventing further quantification of hydrolyzable tannins and their antioxidant capacity. Their results indicated that the defatted material of walnut presented the significant antioxidant capacity (estimated about 332 µmol TEs/g of DM) of this nut; a significant part resulted from insoluble tannins. The influence of walnut oil on the complete antioxidant activity of walnut (FRAP and ABTS assays) was reported to be less than 5%. Also, it was detected that oil could affect the measurement of the antioxidant activities of whole walnut, a fact that could influence the information described in the works. Finally, the authors concluded that it is necessary to determine the antioxidant capacity of oil and defatted material separately.

The kernel and husk portions of walnut fruit have been received increasing attention by different research groups (Table 2) and, thus, there are many reports on the antioxidant content, especially TPC and TFC. For better interpretation, all the obtained values in Table 2 for the TPC of the walnut kernel were converted to mg/g based on DW, fresh weight (FW) and extract. As has been summarized in Table 3, the TPC of the walnut kernel is in the range of 1.45–18.61 mg/g of DW, while it contains 7.35–86.67 mg/g of FW. Besides, the content of total phenolic in the prepared walnut kernel extract is 25.06–166 mg/g. For a better comparison of the TFC values measured for the walnut kernel, the data were also converted to mg/g (For further details, refer to Table 3). The TFC of the walnut kernel is 0.9–7.44 mg/g of DW. There is no information regarding the content of flavonoids in the fresh walnut kernel. However, 10–20 mg/g for the TFC has been reported for the obtained extract derived from the walnut kernel. It is clear that the TPC and TFC of the extract are higher than that of the values reported for the dried or fresh walnut kernel. It seems that the execution extraction procedure using certain solvents enables the detection of the higher concentration of antioxidant content for walnut kernel extract compared to its DW or FW.

#### 5.1.2. Skin

According to the performed studies, the skin or seed coat surrounding the kernel contains high amounts of antioxidant compounds. For example, twelve genotypes of *J. regia* L., six with red skin and another six with light yellow skin were selected for investigating some nutritional and functional components of the kernel and skin [66]. Significantly higher levels of TPC and TFC in yellow skins than in red skins were found, while no significant differences between the two groups of genotypes were reported in kernels. Authors indicated that walnuts could be considered as a good source of dietary antioxidants, and most of them are concentrated in the seed coat. It has been documented that despite the beneficial effects of walnut, its phenolics may adversely influence the protein solubility [62]. For this reason, the kernels of three Argentinian walnut varieties, Chandler, Criolla, and Franquette were selected to evaluate the effect of skin removal on the solubility of walnut kernel proteins [154]. Higher TPC for skin extracts was obtained, and their extracts had stronger antioxidant activity than kernel flour extracts.

Different TPC values obtained in studies related to the skin of the walnut kernel are tabulated in Table 2. For better interpretation, all of them were also changed to mg/g in order to obtain a concentration range for the TPC and to compare the values. As has been presented in Table 3, for both DW and the studied extracts, the skin portion of walnut fruit contains a high amount of TPC (52.05–279.3 and 490 mg/g, respectively). In the case of TFC, only total flavonoid for the dry skin of the walnut fruit has been reported (0.5–10.96 mg/g). The most crucial point is that the content of flavonoids in both kernel and skin, particularly in the skin, is reported to be low, demonstrating that walnut kernel with its seed coat contains phenolics mostly of the non-flavonoid type, which belong to the phenolic acids and hydrolyzable tannins.

#### 5.1.3. Shell

In an investigation, the antioxidant activities and xanthine oxidase inhibition effects of husk, shell and defatted kernel of walnut fruit, as well as the stem and leaf, were studied. Surprisingly, 14.81 g GAEs/100 g dry extract (DE) as the highest TPC were recorded for the obtained extract from the shell portion followed by leaf extract, stem extract, defatted kernel extract, and green husk extract [158]. In another investigation, among different solvents including water, chloroform, methanol, ethanol, ethyl acetate and n-butanol, the highest TPC and TFC were reported for the walnut shell extracted with ethyl acetate [140]. In another study by the same authors [141], among different examined solvents (water, chloroform, methanol, ethanol, ethyl acetate, and n-butanol), the highest TPC (200.40 mg GAEs/g extract) and TFC (80.40 mg QEs/g extract) were achieved with ethyl acetate. Additionally, they investigated the inhibitory effect of extracts on the adipogenesis of 3T3-L1 preadipocytes and found that the methanol extract had the highest inhibitory activity under the concentration of 500 μg/mL.

The reported data related to TPC and TFC of the shell portion of walnut fruit summarized in Table 2 are also expressed as mg/g. As illustrated in Table 3, it can be observed that the TPC for the shell was 18.4 and 68.34–200 mg/g in the DW and the prepared extract, respectively. The content of total flavonoid values for the walnut shell was also changed to mg/g, and it was found that it contains 4.86 and 80.40–162.5 mg/g of DW and the extract, respectively. Both TPC and TFC have not been determined in the fresh walnut shell. These reported values indicated that the obtained extracts derived from walnut shell have a higher content of phenolic and flavonoid constituents.

#### 5.1.4. Husk

The TPC of walnut husk was obtained in numerous studies; however; the values were reported in different units, as shown in Table 2. For this reason, all of them were converted to the unit of mg/g for getting the exact amounts of total phenolics in the walnut husk (Table 3). The TPC of walnut is estimated to be 6.27–36.10 for the DW, while the TPC was in the range of 50.18–166.44 mg/g for the considered extracts. Additionally, the values obtained for the content of total flavonoids in walnut green husk were considered as mg/g, and it was determined to be 0.7–12.22 and 22.91–65.2 mg/g on the DW and the prepared extracts, respectively. The fresh walnut husk has not been studied for its TPC and TFC determination.

### 5.2. Other Parts of the Tree

In addition to different portions of fruit, some other parts of the walnut tree have recently gained considerable interest. For this reason, many studies have reported the total phenolic and flavonoid contents of other parts of the walnut tree, especially the data related to the leaf are considerable. However, extensive research on TFOC and TCTC are rare, or have not been reported for a number of other constituents such as TFAC and THTC (see Table 4 for further details).

#### 5.2.1. Leaf

Among different parts of the walnut tree, the leaf part is the most considered material, confirming its broad application from ancient times traditional folk medicine of some countries. Extracts from walnut leaf were thoroughly evaluated in numerous studies, and their antioxidant content has been determined. Salimi, et al. [165] examined some solvents with different polarities including hexane, chloroform, ethyl acetate, methanol and ethanol for the evaluation of the cytotoxicity of walnut leaf extract on human cancer cell lines. Using colorimetric methods, they reported that the obtained methanolic extract presented the highest amount of TPC, TFC, and TCTC (120.28 ± 2.32, 59.44 ± 0.87, and 227.00 ± 4.91 mg/g DE, respectively). In this study, the authors prepared total extract using ethanol from young and mature leaves of walnut. They found that the contents of the phenolic compounds in young leaves were significantly higher than that of their contents in the mature leaves. It has been recommended that walnut leaves with higher phenolic content should preferentially be collected in July and early September [186]. In this study, the TPC of twelve different cultivars of walnut leaves, over eight distinct harvest times (June 1st to September 15th), was studied. Phenolic compound extracts from walnut leaves were prepared using the UAE and RSM methods by Nour, et al. [104]. In this study, they tried to establish the optimum conditions and the maximum predicted TPC, under the optimized conditions of 61% ethanol concentration, 51.28 min extraction time and the 4.96 *v*/*w* liquid-to-solid ratio was 10,125.4 mg GAEs/l, while 2925 mg quercetin equivalents (QEs)/l as maximum TFC was achieved by using ethanol with 67.83% concentration, *v*/*w* liquid-to-solid ratio of 4.96 and 49.37 min of extraction time. Under these conditions, the experimental results were close to the predicted values calculated from the polynomial response surface model equation. Evaluating some solvents for the extraction of phenolics from walnut husk showed the high efficiency of the ethanol or methanol. It has been elucidated that among different tested solvents (water, methanol, ethanol, acetone, and benzene), the obtained methanolic extract from walnut leaves contained the highest TPC (94.39 ± 5.63 mg GAEs/g extract) as compared to aqueous extract (27.92 ± 1.40 mg GAEs/g extract) [145]. In another study, the antioxidant and antimicrobial activities of the prepared ethanol and water extracts from the leaves of three plants, namely *P. aphylla*, Persian walnut, and oleander, were evaluated. The results showed that in all assays, the ethanol extracts had the highest amount of total phenolics and flavonoids, as well as highest antioxidant and antimicrobial activities when compared to the water extracts [149].

As has been shown in Table 4, the content of total phenolics of the walnut leaf was reported in different units. Accordingly, all the summarized data for the TPC and TFC of walnut leaf in Table 4 were converted to mg/g (Table 3), and it was seen that the leaf part in the dried form has considerable amounts of TPC ranging from 34 to 194 mg/g. Besides, the extracts derived from walnut leaf show higher amounts of phenolics. For the TFC of the walnut leaf, a high amount of flavonoids in both DW and the investigated extracts (20 and 20.17–149 mg/g, respectively) was found. As has been shown in Table 3, the content of phenols and flavonoids have not been reported for the fresh walnut leaf.

#### 5.2.2. Shoot

As has been presented in Table 3 and Table 4, only the TPC and TFC of the fresh shoot have been studied [81,82]. There are no data on the chemical contents of the dried or the prepared extracts of the shoot.

#### 5.2.3. Bark

Walnut bark is another portion of the tree which has been considered in some studies for the evaluation of its different properties, including antioxidant content (see Table 3 for more details). Interestingly, the dried walnut bark has significant amounts of TPC compared to its prepared extracts (34.83–311 and 9.8 mg/g, respectively, refer to Table 4). The TPC of fresh walnut bark has not been considered. Moreover, there is no information regarding the TFC or extract of dried or fresh walnut bark.

#### 5.2.4. Stem

The values of TPC in Table 4 reported for the stem of the walnut tree were also changed to mg/g, as shown in Table 3. The investigated extracts derived from walnut stem contain extraordinary amounts of phenolic compounds. Accordingly, its TFC is also considerable. The TPC and TFC of dried and also fresh bark have not been determined.

#### 5.2.5. Root

The prepared ethanolic extract from the walnut root was chemically characterized by gas chromatography–mass spectrometry (GC-MS), and the phytochemical experiments showed the presence of phenols, alkaloids, steroids, saponins, and tannins. In the GC-MS results, a total of 40 various compounds were identified [21]. However, no detailed study on the the content of phenolic and flavonoid chemicals of the walnut root has been reported.

## 6. Total Antioxidant Activity

### 6.1. Fruit

The antioxidant activity of different parts of walnut fruit was evaluated by some spectroscopic-based methods such as total antioxidant activity (TAA), reducing power (RP), FRAP, the oxygen radical absorbance capacity (ORAC), and lipid peroxidation inhibition in different investigations (Table 5). For more details on the mechanism of the antioxidant activity methods, readers are prompted to consider the review paper published by Craft, et al. [213]. Unfortunately, The obtained results in different studies have been reported in different units, i.e., mmol Fe^2+^, EC_50_, EC_25_, SC_50_, IC_50_, mg FeSO_4_ and the absorbance (Abs) at specific wavelengths and, thus, the exact antioxidant capacity value for each part of the fruit cannot be determined from the values summarized in Table 5. Interestingly, the highest phenolic content and the best antioxidant activity for ORAC value was 3423.44 ± 142.52 µmol TEs/g) for the shell extract of walnut [158]. In this study, the authors evaluated the antioxidant activities and xanthine oxidase inhibition properties of different parts of fruit (husk, shell, and defatted kernel) and the stem and leaf portions of walnut. It was shown that the antioxidant capacities of all extracts evaluated by FRAP and ORAC assays were in agreement with the content of total phenolic except for defatted kernel extract.

### 6.2. Other Parts of the Tree

In addition to different parts of walnut fruit, the antioxidant capacity of other parts of the tree by the preparation of extracts was also evaluated and reported (Table 6). TAA, RP, FRAP, ORAC, and lipid peroxidation inhibition are the main methods used for the antioxidant capacity evaluation of the obtained extracts. Unfortunately, all the data regarding the antioxidant activity of different parts of the walnut tree are restricted to several values, and the obtained values are reported in different units.

## 7. Radical Scavenging Activity

### 7.1. Fruit

#### 7.1.1. Reactive Oxygen Species (ROS)

The highly reactive molecules derived from oxygen molecules are known as ROS. Recently, ROS have received increasing interest because they are produced in the body as a result of aerobic metabolism, and their overproduction is related to some diseases initiation and progress. Additionally, a connection between ROS generation and the onset of senescence process in the human body has been well evidenced. Hydroxyl radical (HO^•^), superoxide radical (O^•−^_2_), peroxyl radical (ROO^•^) and hydrogen peroxide (H_2_O_2_) are the main ROS molecules. In recent years, the potent radical scavenging activity of the extracts against ROS can be devalued under in vitro conditions. The obtained extracts from different parts of walnut fruit have been evaluated for their abilities in the scavenging of different types of ROS radicals (Table 7). For different parts of walnut fruit, only the hydrogen peroxide radical scavenging of kernel and husk extracts was tested [182]. In the case of superoxide radical scavenging, the antiradical activities of extracts derived from different parts of walnut fruit have been comprehensively investigated in several research studies [67,140,182]. Among different parts of extracts derived from walnut fruit, only the radical scavenging activity for the shell extract was reported [140]. There are no reports on the peroxyl radical scavenging activity of walnut fruit.

#### 7.1.2. Reactive Nitrogen Species (RNS)

RNS are reactive chemicals containing nitrogen and derived from nitric oxide. Nitric oxide (^•^NO) and peroxynitrite anion (ONOO^−^) are the main RNS molecules. As has been shown in Table 7, the radical scavenging activity of the prepared extracts from all parts of walnut fruit especially, kernel and husk, were tested against NO radical [67,71,103,182]. However, their potential activities in the scavenging of peroxynitrite have not been evaluated.

#### 7.1.3. DPPH Radical

DPPH^•^ has been investigated since the 1950s for its use as an organic colorimetric reagent [214]. In the last two decades, the DPPH^•^ test has emerged as a method for analyzing phenols in plants and food products derived from plants [215]. Braude, et al. [216] observed that DPPH^•^ behaves as an antioxidant in the hydrogen atom transfer (HAT) mechanism. A concentration-dependent antioxidant potential was reported in reducing power and DPPH assays, with EC_50_ values lower than 1 mg/mL for all the tested aqueous extracts of walnut green husks among five different cultivars (Table 7) [73]. The highest ability to scavenge DPPH, hydroxyl and superoxide radicals were registered for ethyl acetate extract (EC_50_ = 81.03 μg/mL), methanol extract (EC_50_ = 131.35 μg/mL) and chloroform extract (EC_50_ = 176.35 μg/mL), respectively. In this research, various solvents such as water, chloroform, methanol, ethanol, ethyl acetate, and n-butanol have been tested for analyzing the extraction yields, total flavonoids content and antioxidant properties of the walnut shell [140]. Additionally, the methanol extract showed the greatest total antioxidant activity and reducing power.

#### 7.1.4. ABTS Radical Cation

ABTS radical cation scavenging activity is also commonly employed to measure the antioxidant capacities of the prepared extracts from different parts of walnut fruit. In this way, the ABTS radical cation scavenging capacity of kernel and husk has been evaluated, but information regarding the ABTS radical cation scavenging of skin and shell is unknown (Table 7).

### 7.2. Other Parts of the Tree

#### 7.2.1. ROS

Among other parts of the walnut tree, only the radical scavenging activity of the extracts obtained from leaf and bark has been examined against ROS radicals (Table 8). Additionally, the radical scavenging potential of any parts of walnut tree extracts for the peroxyl radical has not been reported.

#### 7.2.2. RNS

Walnut leaf extract is the only part of the tree which has been investigated for its capability to scavenge NO and ONOO (Table 8). The preparation of extracts from other parts of the walnut tree such as shoot, branch, bark, and even root has not been reported.

#### 7.2.3. DPPH Radical

Leaf and bark are the main parts of the walnut tree examined for DPPH radical scavenging capacity. For example, an ethanol:water (4:6) extract from walnut leaves was investigated for its putative in vitro scavenging properties on ROS including hydroxyl, superoxide, peroxyl, and hydrogen peroxide radicals and RNS including nitric oxide and peroxynitrite anion [163]. The extract showed a strong scavenging capacity versus all the studied reactive molecules, all of the IC_50_ values being calculated at the µg/mL level. IC_50_s for the O^•−^_2_ and H_2_O_2_ were 47.6 ± 4.6, 383 ± 17 µg/mL, respectively. The ORAC value achieved for ROO^•^ was 2.17 ± 0.22 µmol TEs/mg extract. The IC_50_s for NO^•^ and ONOO^−^ were 1.95 ± 0.29 and 1.66 ± 0.10 µg/mL, respectively (Table 8). Their results show that walnut leaf extract can be used as an easily reachable source of natural antioxidants. Moreover, among various investigated solvents (water, methanol, ethanol, acetone, and benzene), the prepared methanolic extract from walnut leaves exhibited the highest DPPH radical scavenging activity followed by the water extract (0.199 ± 0.023 and 2.991 ± 0.740, respectively) [145]. The radical scavenging of extracts prepared from shoot and stem have been tested, but the DPPH radical scavenging of the branch remains unknown.

#### 7.2.4. ABTS Radical Cation

The prepared extracts from other parts of the walnut tree have been tested for their capacity in the scavenging of ABTS radical cation. Only the ABTS radical cation scavenging capacity of bark extract has been reported, but information on the ABTS radical scavenging of other parts such as the leaf, shoot, and brank is not available (Table 8).

## 8. Conclusions

As an ancient herb, various parts of walnut have therapeutic potential or preventive properties. The results of most investigations strongly show that walnut is an excellent source of active chemo-preventive constituents and natural antioxidants. Today, it has received increasing attention because of the highest antioxidant content of walnut among different commonly consumed nuts. The flesh of walnut fruit is placed inside three layers, called the seed coat or skin, the inner shell, and outer green shell cover or husk. Shell and husk are agricultural by-products that are considered as valuable sources of different phenolics or other beneficial compounds produced in large quantities upon the processing of the fruit. Such agricultural by-products are generally used as fuel. The skin, shell, and husk constitute over 70% of the total weight of the walnut fruit and are a readily available source of beneficial compounds.

Walnut green husks can be an excellent source of valuable compounds with antioxidant, antimicrobial activity, and health protection potential. For the effective use of shell and also branch, a waste product from the cultivation of the walnut tree, pyroligneous acids can be prepared and collected by pyrolyzing at higher temperature conditions. The obtained pyroligneous acids from the shell and tree branch have high potential to be industrialized as natural antioxidant, antibacterial and antifungal reagents in the fields of medicine, food industry, and agriculture. The highest levels of phenolic compounds are found to be concentrated in the skin. The results of some research on walnut skin clearly showed the importance of seed coat in the higher antioxidant activity of walnut kernel compared to other nuts. The total antioxidant activity reduced considerably on the removal of the seed coat. Although the skin contributes less than 5% to the walnut fruit weight, its TPC is at least 93–97% higher than that of the entire kernel, showing that skins are a good source of phenolic compounds than whole kernels. The papers discussed here established the antimicrobial potential of walnut bark, thus supporting its application as a preventive remedy in the oral cavity for various microbial diseases (periodontal and caries disease). Walnut leaves could be considered as another good source for the obtainment of polyphenol extracts. In this regard, extracts with a high content of phenolics as dietary supplements, nutraceuticals, and functional food ingredients were prepared using the UAE method.

Phenolic compounds inhibit lipid oxidation by scavenging free radicals, chelating metals, activating antioxidant enzymes, reducing tocopherol radicals and inhibiting enzymes that cause oxidation reactions. The conducted studies may provide the basis for the rapidly growing interest in the use of natural antioxidants and antimicrobials. The difference in the extraction yields could probably be explained by the different methods of extraction and the considered solvents. The selection of a suitable solvent is an essential point for the obtainment of phenolics extracts with high antioxidant activity, which is useful for the development and application of the extracts derived from different parts of walnut. In most studies, dealing with the extraction and evaluation of the antioxidant content of different parts of walnut tree, the prepared extracts using polar solvents (methanol, ethanol or water) exhibited higher phenolic content and antioxidant potential than the extracts prepared by nonpolar solvents, i.e., petroleum ether or n-hexane. This fact can be supported by the importance of solvent polarity and also the solubility of different phenolic compounds. Thus, the solubility and the efficiency of the extraction procedure are considerably affected by the differences in the polarity of the solvents used. Various polar solvents were reported to be the best choice for the extraction of phenolics constituents from different parts of the walnut plant, while the non-polar solvents were found as non-effective solvents and frequently used for the extraction of less polar compounds, such as chlorophylls, carotenoids tocotrienols, and tocopherols. Therefore, the obtained walnut extracts using polar solvents are probably contained phenolics (phenolic acids, flavonoids, and tannins), while the resulting extracts from nonpolar solvents are mostly enriched in non-polar constituents such as tocopherols, tocotrienols, fatty acids and sterols and deficient in phenolic compounds. Methanol, ethanol and the hydroalcoholic are generally suggested as suitable extraction solvents for the preparation of the phenolic extract. Additionally, the differences may be attributed in part to different extraction methods, varieties evaluated, year of harvest, orchard location, processing, and storage. The differences in phenolic compound distribution in different parts of the nut could be the reason for the low content of total phenolic in walnut oil.

Finally, it could be said that recent investigations into the phytochemical composition of skin, shell, husk and even other parts such as leaf, bark, and root have shown that they may contain many potentially beneficial compounds, opening up new possibilities for adding value to agricultural by-products of walnut. Nevertheless, further studies are required to determine more details regarding the individual chemical constituents and the possible effects in order to use them practically in the pharmaceutical, food, and medicine fields.

## Figures and Tables

**Table 1:** The considered solvents for the preparation of extracts from different parts of the walnut tree and its fruit.

No	Solvent		Part	References
1	Water	Fruit	Kernel	[28,69,70,136,137,138,139]
			Shell	[56], [140]^25^, [141]^25^
			Husk	[18,56,73,103,142,143]
		Other parts	Leaf	[78]^25^, [109], [144]^24^, [145,146], [147]^25,^ [148,149], [150]^28^
			Bark	[128], [151]^32^, [152,153]
			Stem	[148]
2	Ethanol	Fruit	Kernel	[28]^9^, [137], [154]^16^, [155]^11^, [156]^11^, [157]^4^, [158]^34^
			Skin	[154]^16^, [159]^35^
			Shell	[24]^4^, [140,141], [158]^34^
			Husk	[18], [103]^4^, [142], [158]^34^, [160]^6^, [161], [162]^4^
		Other parts	Leaf	[104]^30^, [110]^6^, [145], [149], [158]^34^, [162]^4^, [163]^31^, [164]^22^, [165]^23^, [166]^22^, [167]^26^, [168,169], [170]^20^, [171,172], [173]^6^
			Bark	[128,151], [174]^2^
			Stem	[158]^34^
			Root	[21]
3	Methanol	Fruit	Kernel	[27,66,67,131,132,133,134,135,138], [154]^17^, [175], [176]^12^, [177], [178]^12^, [179]^14^, [180]^15^, [181]^12^, [182]
			Skin	[66,67], [154]^16^
			Shell	[56,67,140,141,183]
			Husk	[18,56,67,71], [103]^5^, [131,182], [184,185],
		Other parts	Leaf	[77,112,131,145,146,148,165,185,186], [187]^27^, [188,189,190,191], [192]^21^, [193,194]
			Shoot	[80], [81]^37^, [82]^37^
			Bark	[124,151,152,195,196], [197]^33^, [198]^3^
			Stem	[148]
			Root	[129]
4	Ethyl acetate	Fruit	Kernel	[70]
			Shell	[56,140,141]
			Husk	[18,56,142]
		Other parts	Leaf	[165]
			Bark	[124,152,195]
			Root	[129]
5	Acetone	Fruit	Kernel	[16]^18^, [25]^10^, [46]^18^, [138], [199]^8^, [200]^13^, [201]^7^, [202]^19^,
			Shell	[183]
			Husk	[18], [102]^7^, [203]^7^
		Other parts	Leaf	[145,204]
			Bark	[124]^1^, [151], [152]^1^, [153], [195]^8^
6	Hexane	Fruit	Husk	[18]
		Other parts	Leaf	[165], [205]^29^
			Bark	[196]
			Root	[129]
7	Dichloromethane	Fruit	Shell	[56]
			Husk	[56]
8	Petroleum ether	Fruit	Kernel	[70,131]
			Husk	[131,142]
		Other parts	Leaf	[131]
			Bark	[151]
9	Chloroform	Fruit	Shell	[140,141]
			Husk	[142]
		Other parts	Leaf	[79,165]
			Bark	[151,196]
10	*n*-Butanol	Fruit	Kernel	[68,70]
			Shell	[140,141]
11	Benzene	Other parts	Leaf	[145]
			Bark	[151]
12	Cyclohexane	Other parts	Leaf	[171]

^1^ in diluted form; ^2^ 80% (400 mL ethanol 96% + 100 mL water); ^3^ 70% aqueous acetone; ^4^ 50% aqueous ethanol; ^5^ 50% aqueous methanol; ^6^ 70% ethanol; ^7^ acetone/water (*v/v*, 70/30); ^8^ acetone:water (80:20; *v/v*); ^9^ 96%; ^10^ chilled 80% acetone (1:8, *w/w*); ^11^ ethanol:water (4:1, *v/v*); ^12^ methanol:water (70:30); ^13^ acetone:water (60:40; *v/v*); ^14^ 80% methanol (1:2 *w/v*, 3×); ^15^ methanol/water (60:40, *v/v*); ^16^ ethanol/water (7:3, *v/v*); ^17^ methanol/water (6:4, *v/v*); ^18^ 75% acetone + 25% of 526 µmol/L sodium metabisulfite; ^19^ 100 mM acetate buffer, pH 4.8/acetone (30:70, *v/v*); ^20^ hydroethanolic (20 to 70% *v/v*); ^21^ 80% methanol (*v/v*); ^22^ 80% ethanol; ^23^ ethanol:water (80:20); ^24^ acidified water (pH 2 with HCl); ^25^ boiling water; ^26^ 60% ethanol; ^27^ 60% methanol; ^28^ 20% water (*w/w*) and choline chloride with butyric or phenylpropionic acid (mole ratio 1:2); ^29^ 95% hexane; ^30^ 61% ethanol; ^31^ ethanol:water (4:6); ^32^ distilled water; ^33^ 99.5% methanol; ^34^ 95% ethanol (*v/v*); ^35^ ethanol:water (4:6); ^36^ 50 or 70% ethanol; ^37^ 80% methanol.

**Table 2:** TPC, TFC, TFOC, TFAC, THTC, and TCTC values in different parts of walnut fruit.

No		Parts	Unit	Content	References
1	TPC	Kernel	mg GAEs/50 g FW	802	[46]
			mg GAEs/100 g nut	1625	[16]
			mg GAEs/g extract ^a^	95.06 ± 0.03	[69]
			mg GAEs/g extract ^a^	25.6 ± 4.73	[154]
			mg GAEs/100 g DM	1071 ± 35.00	[207]
			mg GAEs/100 g DM	605	[156]
			mg GAEs/100 g nut	1580 ± 58.0	[25]
			mg GAEs/100 g sample ^a^	515 ± 21	[28]
			mg GAEs/100 g FW	2499 ± 94	[176]
			mg GAEs/g extract	116.22 ± 3.7	[131]
			mg GAEs/g extract	31.66 ± 0.11	[27]
			mg GAEs/g DW	22	[199]
			mg GAEs/g ^a^	35.22 ± 0.75	[137]
			mg GAEs/100 g sample ^a^	145 ± 12.58	[67]
			mg GAEs/g FW	58 ± 3	[208]
			mg GAEs/100 g DM	1404 ± 23	[178]
			mg GAEs/100 g	2464 ± 22	[200]
			mg GAEs/g extract	166 ± 0.8	[201]
			mg GAEs/g FW	86.67 ± 0.09	[209]
			mg GAEs/100 g FW	1456 ± 235	[133]
			mg GAEs/kg FW ^b^	7351.65 ± 564.60	[180]
			g GAEs/100 g DE	10.43 ± 0.18	[158]
			mg GAEs/100 g DW ^b^	1861.11 ± 535.50	[66]
			mg GAEs/g ^a^	50.3 ± 0.7	[181]
			mg GAEs/g extract ^a^	86.56 ± 2.00	[139]
			mg GAEs/100 g extract	3490 ± 4.9	[132]
			mg GAEs/g ^a^	66.55 ± 4.98	[182]
		Skin	mg GAEs/g extract ^a^	490 ± 27.3	[154]
			mg GAEs/100 g sample ^a^	5205 ± 127.61	[67]
			mg GAEs/100 g DW ^b^	27903.86 ± 5980.09	[66]
		Shell	mg GAEs/100 g sample ^a^	1804 ± 42.02	[67]
			mg GAEs/g extract ^a^	200.40 ± 0.70	[141]
			g GAEs/100 g DE	14.81 ± 0.50	[158]
			mg GAEs/g DE ^a^	68.34 ± 5.9	[56]
		Husk	mg/100 g DW	1526 ± 111.00	[22]
			mg GAEs/g extract ^a^	74.08 ± 0.02	[73]
			mg GAEs/g extract	50.18 ± 2.7	[131]
			mg GAEs/g extract ^a^	108.11 ± 4.6	[71]
			mg GAEs/100 g sample ^a^	3610 ± 55	[67]
			mg GAEs/g extract ^a^	84.46 ± 2.96	[103]
			mg GAEs/DS.	7.23	[161]
			mg GAEs/L extract ^a^	4610 ± 262.73	[160]
			mg/g	46.88	[142]
			g GAEs/100 g DE	8.74 ± 0.33	[158]
			mg GAEs/g sample	6.27	[18]
			mg GAEs/g extract ^a^	166.44 ± 1.87	[203]
			mg GAEs/g DE ^a^	58.66 ± 0.37	[56]
			mg GAEs/g DE ^a^	95.2 ± 6.29	[102]
			mg GAEs/g ^a^	122.26 ± 1.34	[182]
2	TFC	Kernel	mg CEs/100 g nut ^c^	744.8 ± 93.3	[25]
			mg QEs/g ^a^	20.02 ± 0.12	[137]
			mg CEs/100 g sample ^a^	93.07 ± 7.86	[67]
			mg QEs/100 g DW ^b^	119.80 ± 27.82	[66]
			mg CEs/g ^a^	13.57± 2.27	[182]
		Skin	mg CEs/100 g sample ^a^	1096 ± 42.27	[67]
			mg QEs/100 g DW ^b^	51.94 ± 7.95	[66]
		Shell	mg CEs/100 g sample ^a^	486 ± 37.22	[67]
			mg REs/g extract ^a^	162.54 ± 1.61	[140]
			mg QEs/g extract ^a^	80.40 ± 0.55	[141]
		Husk	mg QEs/g extract ^a^	22.91 ± 1.1	[71]
			mg CEs/100 g sample ^a^	1064 ± 81	[67]
			mg QEs/L extract ^a^	423.97 ± 10.37	[160]
			mg TAEs/g	12.88	[142]
			mg REs/g sample ^a^	0.71	[18]
			mg CEs/g DE	65.2 ± 5.53	[102]
			mg CEs/g ^a^	49 ± 3.17	[182]
3	TFOC	Kernel	mg QEs/g extract ^a^	2.3 ± 0.1	[201]
		Husk	mg QEs/g extract ^a^	2.3 ± 0.1	[203]
			mg REs/g extract	21.2 ± 5.53	[102]
4	TFAC	Kernel	mg CEs/g extract	34.7 ± 1.1	[201]
			mg/kg FW ^b^	582.10 ± 35.49	[180]
		Husk	mg CEs/g extract	34.7 ± 0.8	[203]
5	THTC	Kernel	mg GAEs/100 g DM	1700 ± 221	[207]
			mg/kg FW ^b^	1999.50 ± 48.11	[180]
6	TCTC	Kernel	g/100 g DM	0.38 ± 0.026	[207]
			mg CYEs/g extract	59.8 ± 2.0	[201]
		Husk	mg CEs/g sample ^a^	3.18	[18]
			mg LEs/g extract	5.8	[102]

^a^ indicating the highest reported value; ^b^ indicating the mean of the reported value; ^c^ Indicating the total of free and bound form. Abbreviations: TPC: total phenolic content; TFC: total flavonoid content; TFOC: total flavonol content; TFAC: total flavanol content; THTC: total hydrolyzable tannin content; TCTC: Total condensed tannin content; GAEs: gallic acid equivalents; FW: fresh weight; DM: dry matter; DE: dry extract; DW: dry weight; QEs: quercetin equivalents; CEs: Catechin equivalents; REs: rutin equivalents; TAEs: tannic acid equivalents; CYEs: cyanidin equivalents; LEs: leucocyanidin equivalents.

**Table 3:** The TPC and TFC values for different parts of the walnut tree and its fruit.

		Content (mg/g)					
		TPC			TFC		
		DW	FW	Extract	DW	FW	Extract
Fruit	Kernel	1.45–18.61	7.35–86.67	25.06–166	0.9–7.44	-	10–20
	Skin	52.05–279.3	-	490	0.5–10.96	-	-
	Shell	18.04	-	68.34–200	4.86	-	80.40–162.5
	Husk	6.27–36.10	-	50.18–166.44	0.7–12.22	-	22.91–65.2
Other parts of tree	Leaf	34–194	-	33.67–410	20	-	20.17–149
	Shoot	-	46.53	-	-	3.83	-
	Bark	34.83–311	-	9.8	-	-	-
	Stem	-	-	71–117	-	-	12.14

- not quantified. Abbreviations: TPC: total phenolic content; TFC: total flavonoid content; DW: dry weight; FW: fresh weight.

**Table 4:** TPC, TFC, TFOC, and TCTC values in other parts of the walnut tree.

No		Parts	Unit	Content	References
1	TPC	Leaf	g/kg DB ^a^	34.5	[77]
			mg/kg DB ^a^	64596.4	[78]
			mg GAEs/g LE ^a^	270 ± 3.00	[163]
			mg GAEs/g extract	94.39 ± 5.63	[131]
			mg GAEs/g	410 ± 14.43	[164]
			mg GAEs/g extract ^a^	33.67 ± 3.06	[186]
			mg GAEs/100 g DS	3704 ± 88	[187]
			mg GAEs/g DE	267.30 ± 2.19	[165]
			% DW extract ^a^	80.3 ± 0.5	[192]
			mg GAEs/g extract	94.39 ± 5.63	[145]
			g GAEs/100 g DE	8.74 ± 0.33	[158]
			mg GAEs/L extract	10125.4	[104]
			mg GAEs/g DE	103.33	[148]
			mg QGEs/g DS	194.9	[172]
			mg GAEs/g DW ^a^	46.47 ± 0.89	[193]
			mg GAEs/g DW	52.82 ± 0.73	[149]
		Shoot	mg GAEs/g of FW ^a^	46.534	[82]
		Bark	mg GAEs/g extract	9.8	[126]
			^4^mg GAEs/g DW	34.833	[195]
			mg GAEs/g DW	311.5	[152]
		Stem	g GAEs/100 g DE	11.70 ± 0.05	[158]
			mg GAEs/g DE	71.51	[148]
2	TFC	Leaf	mg REs/g	330 ± 12.21	[164]
			mg QEs/g DE	149.00 ± 2.55	[165]
			mg QEs/100 g DS	3117 ± 84	[187]
			mg QEs/L extract	2952	[104]
			mg QEs/g DE	20.17	[148]
			mg QEs/g	28.48 ± 0.12	[193]
			mg GAEs/g DW ^a^	20.06 ± 1.07	[149]
		Shoot	mg QEs/g FW ^a^	3.837	[82]
		Stem	mg QEs/g DE	12.14	[148]
3	TFOC	Leaf	mg REs/g	270 ± 22.33	[164]
			mg CAEs/g DS	66	[172]
			mg QEs/g	21.76 ± 0.25	[193]
4	TCTC	Leaf	mg LEs/g DE	950.56 ± 4.50	[165]
			µg CEs/mg	5.37 ± 0.07	[146]
		Shoot	mg CEs/g FW ^a^	47.983	[82]
		Bark	mg CEs/g DW ^a^	16.167	[195]
			mg CEs/g DW	38.5	[152]

^a^ indicating the highest reported value. Abbreviations: TPC: total phenolic content; TFC: total flavonoid content; TFOC: total flavonol content; TCTC: Total condensed tannin content; DB: dry basis; GAEs: gallic acid equivalents; LE: lyophilized extract; DS: dry sample; DE: dry extract; DM: dry matter; FW: fresh weight; REs: rutin equivalents; QEs: quercetin equivalents; DW: dry weight; CAEs: chlorogenic acid equivalents; LEs: leucocyanidin equivalents; CEs: Catechin equivalents.

**Table 5:** TAA, RP, FRAP, ORAC, and lipid peroxidation inhibition values in different parts of walnut fruit.

No		Parts	Unit	Content	References
1	TAA	Kernel	mg TEs/L ^b^	7850 ± 337.55	[160]
2	RP	Kernel	EC_50_ (mg/mL) ^a^	0.16	[69]
			EC_50_ (mg/mL)	11.03 ± 0.36	[27]
			Abs at 700 nm ^c^	0.13 ± 0.0	[67]
			IC_50_ (μg/mL)	238.38 ± 2.90	[158]
			EC_50_ (mg/mL) ^a^	0.2 ± 0.001	[139]
		Skin	Abs at 700 nm ^c^	1.25 ± 0.02	[67]
		Shell	Abs at 700 nm ^c^	0.56 ± 0.02	[67]
			IC_50_ (μg/mL)	115.86 ± 2.05	[158]
		Husk	EC_50_ (mg/mL) ^a^	0.5	[73]
			Abs at 700 nm ^c^	0.44 ± 0.01	[67]
			EC_50_ (mg/mL) ^a^	0.95 ± 0.02	[103]
			IC_50_ (μg/mL)	150.33 ± 3.60	[158]
3	FRAP	Kernel	µmol TEs/g DM	154.88 ± 15.26	[207]
			µmol Fe^2+^/100 g FW	13.24 ± 1.99	[133]
			mg FeSO_4_ /g ^b^	302 ± 12	[181]
			µM Fe^2+^/g extract	522 ± 13.40	[139]
		Shell	mmol Fe^2+^/g DE	2202.29 ± 0.5	[56]
		Husk	mmol Fe^2+^/g DS.	0.33–0.46	[161]
			Abs at 593 nm	0.024–0.509	[18]
			µmol TEs/g extract	896 ± 18	[203]
			mmol Fe^2+^/g DE	1220 ± 0.6	[56]
4	Lipid peroxidation inhibition	Kernel	EC_25_ (mg/mL) ^a^	1.56	[69]
		Husk	EC_25_ (mg/mL) ^a^	1.27	[73]
			SC_50_ (μg/mL)	120 ± 2.1	[102]
5	ORAC	Kernel	µmol TEs/g DM	187.49 ± 25.31	[207]
			µmol TEs/g extract	1481.21 ± 14.68	[158]
		Shell	µmol TEs/g extract	3423.44 ± 142.52	[158]
		Husk	µmol TEs/g extract	2079.77 ± 90.13	[158]

^a^ indicating the lowest reported value; ^b^ indicating the highest reported value; ^c^ Indicating the mean of reported values. Abbreviations: EC_50_: effective concentration of 50%; Abs: absorbance; IC_50_: inhibitory concentration of 50%; DM: dry matter; FW: fresh weight; DE: dry extract; EC_25_: Effective concentration of 25%; SC_50_: Scavenging capacity of 50%; TEs: Trolox equivalents.

**Table 6:** TAA, RP, FRAP, and lipid peroxidation inhibition values in different parts of the walnut tree.

No		Parts	Unit	Content	References
1	TAA	Shoot	mg VCEs/100 g DW	2872.4 ± 28.26	[82]
		Bark	mg GAEs/g DW ^a^	329	[195]
			mg GAEs/g of DW ^a^	420.66	[152]
2	RP	Leaf	EC_50_ (mg/mL) ^a^	0.192	[78]
			IC_50_ (μg/mL)	121.72 ± 5.18	[158]
			EC_50_ (μg/mL)	1.81 ± 0.11	[149]
		Shoot	50% Abs (mg/mL) ^a^	0.374 ± 0.01	[82]
		Bark	EC_50_ (μg/mL) ^a^	99	[195]
			EC_50_ (μg/mL) ^a^	220	[152]
		Stem	IC_50_ (μg/mL)	134.50 ± 3.38	[158]
3	FRAP	Leaf	µM Fe^2+^/g FW	418.92-1067.94	[193]
4	β- carotene bleaching test	Leaf	EC_50_ (mg/mL) ^a^	0.742	[78]
			Inhibition (%)	84.62 ± 2.85	[146]
		Bark	IC_50_ (μg/mL) ^a^	280	[195]
			IC_50_ (μg/mL) ^a^	730	[152]
		Branch	IC_50_ (μg/mL) ^a^		
5	ORAC	Leaf	µmol TEs/mg extract	2.17 ± 0.22	[163]
			µmol TEs/g extract	2543.50 ± 90.10	[158]
		Stem	µmol TEs/g extract	2540.63 ± 121.01	[158]

^a^ indicating the lowest reported value; ^b^ indicating the highest reported value. Abbreviations: VCEs: Vitamin C equivalents; GAEs: gallic acid equivalents; DW: dry weight; EC_50_: effective concentration of 50%; IC_50_: inhibitory concentration of 50%; Abs: absorbance; FW: fresh weight; BHTEs: butyl hydroxytoluene equivalents; TEs: Trolox equivalents.

**Table 7:** The antiradical activity of extracts of different parts of walnut fruit against various ROS, RNS, and other radicals.

No	Free Radicals		Plant Part	Unit	Content	References
1	ROS	H_2_O_2_	Kernel	Scavenging (%) ^b^	239.80 ± 2.00	[182]
			Husk	Scavenging (%) ^b^	236.86 ± 3.40	[182]
		O_2_^•–^	Kernel	Scavenging (%) ^c^	63.89 ± 0.100	[67]
				Scavenging (%) ^b^	98.48 ± 4.2	[182]
			Skin	Scavenging (%) ^c^	128.48 ± 2.12	[67]
			Shell	Scavenging (%) ^c^	108.12 ± 2.04	[67]
				EC_50_ (µg/mL) ^a^	173.41 ± 3.56	[140]
			Husk	Scavenging (%) ^c^	244.76 ± 1.52	[67]
				Scavenging (%) ^b^	98.48 ± 7.20	[182]
		HO^•^	Shell	EC_50_ (µg/mL) ^a^	97.32 ± 3.29	[140]
2	RNS	^•^NO	Kernel	Scavenging (%) ^c^	9.87 ± 1.82	[67]
				Scavenging (%) ^b^	54.68±1.56	[182]
			Skin	Scavenging (%) ^c^	68.50 ± 1.97	[67]
			Shell	Scavenging (%) ^c^	70.26 ± 2.19	[67]
			Husk	IC_50_ (μg/mL) ^a^	141 ± 0.4	[71]
				Scavenging (%) ^c^	69.18 ± 1.57	[67]
				Scavenging (%) ^b^	60.45	[103]
				Scavenging (%) ^b^	31.35 ± 1.70	[182]
3	Other radicals	DPPH^•^	Kernel	ppm BHTEs	92.6 ± 39.5	[154]
				EC_50_ (mg/mL)	0.15	[69]
				EC_50_ (g/DM/g DPPH)	4.05 ± 0.21	[207]
				EC_50_ (mg/mL extract)	0.143 ± 0.020	[131]
				µmol TEs/g FW	120 ± 10	[176]
				Scavenging (%) ^c^	1.2 ± 0.10	[67]
				µmol TEs/g sample	191 ± 4.2	[178]
				mmol TEs/100 g sample	25.7 ± 2.1	[200]
				mmol TEs/100 g DW	20.42 ± 3.84	[66]
				Scavenging (%) ^b^	40 ± 1	[181]
				EC_50_ (mg/mL)	0.19 ± 0.02	[139]
				Scavenging (%) ^b^	27.48 ± 1.01	[182]
			Skin	ppm BHTEs	286 ± 2.72	[154]
				Scavenging (%) ^c^	73.33 ± 1.18	[67]
				Scavenging (%) ^b^	92.8 ± 0.4	[159]
				mmol TEs/100 g DW	303.01 ± 59.15	[66]
			Shell	Scavenging (%) ^c^	7.19 ± 0.11	[67]
				EC_50_ (µg/mL) ^a^	81.03 ± 2.31	[140]
				IC_50_ (μg/mL) ^a^	10.3 ± 0.8	[56]
			Husk	EC_50_ (mg/mL)	0.35	[73]
				EC_50_ (mg/mL extract)	0.412 ± 0.025	[131]
				IC_50_ (μg/mL) ^a^	122 ± 4.5	[71]
				Scavenging (%) ^c^	12.83 ± 0.08	[67]
				EC_50_ (mg/mL)	0.33 ± 0.02	[103]
				IC_50_ (μg/mL) ^a^	33.98	[161]
				IC_50_ (μg/mL) ^a^	54.9	[18]
				IC_50_ (μg/mL) ^a^	114 ± 1.4	[56]
				SC_50_ (μg/mL)	85 ± 1.6	[102]
				Scavenging (%) ^b^	89.81 ± 1.21	[182]
		ABTS^•+^	Kernel	µmol TEs/100 g DW	7713 ± 176	[28]
				mmol TEs/100 g DW ^a^	0.2	[156]
				mmol TEs/100 g sample	21.4 ± 2	[200]
			Husk	IC_50_ (μg/mL) ^a^	324.8	[18]
				^6^µmol TEs/g extract	1251 ± 16	[203]

^a^ indicating the lowest reported value; ^b^ indicating the highest reported value; ^c^ indicating the mean of reported values. Abbreviations: EC_50_: effective concentration of 50%; IC_50_: inhibitory concentration of 50%; BHTEs: butyl hydroxytoluene equivalents; DM: dry matter; FW: fresh weight; TEs: Trolox equivalents; DW: dry weight; SC_50_: scavenging capacity of 50%.

**Table 8:** The antiradical activity of the extracts prepared from different parts of the walnut tree against various ROS, RNS and other radicals.

No	Free Radicals		Plant Parts	Unit	Content	References
1	ROS	H_2_O_2_	Leaf	IC_50_ (μg/mL)	383 ± 17	[163]
				EC_50_ (μg/mL)	4.30 ± 0.06	[149]
			Bark	IC_50_ (mg/mL)	1.2	[126]
		O_2_^•–^	Leaf	IC_50_ (μg/mL)	47.6 ± 4.6	[163]
				mg TEs/g extract	86.29 ± 2.00	[146]
			Bark	IC_50_ (mg/mL)	0.885	[126]
				IC_50_ (μg/mL)	70	[195]
		HO^∙^	Leaf	Scavenging (%)	62.98 ± 7.48	[146]
2	RNS	^∙^NO	Leaf	IC_50_ (μg/mL)	1.95 ± 0.29	[163]
				Scavenging (%)	65.85 ± 0.99	[146]
		ONOO^∙^	Leaf	IC_50_ (μg/mL)	1.66 ± 0.10	[163]
3	Other radicals	DPPH^∙^	Leaf	EC_50_ (mg/mL) ^a^	0.151	[78]
				EC_50_ (mg/mL extract)	0.199 ± 0.023	[131]
				% ^b^	70.8	[192]
				EC_50_ (mg/mL extract)	0.199 ± 0.023	[145]
				mmol TEs/L extract	56.84 ± 1.88	[104]
				IC_50_ (mg/mL)	0.244	[148]
				% ^b^	73.5	[193]
				EC_50_ (μg/mL)	7.49 ± 0.05	[149]
			Shoot	EC_50_ (mg/mL) ^a^	0.312 ± 0.01	[82]
			Bark	IC_50_ (mg/mL)	0.582	[126]
				IC_50_ (μg/mL)	3	[195]
				IC_50_ (μg/mL) ^a^	4.8	[152]
			Stem	IC_50_ (mg/mL)	0.343	[148]
		ABTS^•+^	Bark	IC_50_ (mg/mL)	0.601	[126]

^a^ indicating the lowest reported value, ^b^ indicating the highest reported value. Abbreviations: EC_50_: effective concentration of 50%; IC_50_: inhibitory concentration of 50%; TEs: Trolox equivalents.
